# Regulation of the PKD2 channel function and associated disease phenotypes by RASSF4

**DOI:** 10.1038/s42003-026-10242-8

**Published:** 2026-05-15

**Authors:** Rui Tian, Wanyi Fang, Wenbin Yuan, Shi Li, Yixin Wu, Xueying Dong, Wei Liu, Jinghua Kong, Xiaoling Deng, Rui Zhang, Hao Lyu, Shuai Xiao, Dong Guo, Qi Zhang, Declan William Ali, Marek Michalak, Cefan Zhou, Jingfeng Tang, Xing-Zhen Chen

**Affiliations:** 1 National “111” Center for Cellular Regulation and Molecular Pharmaceutics, School of Life and Health Sciences, Hubei University of Technology, Wuhan, China; 2 Membrane Protein Disease Research Group, Department of Physiology, Faculty of Medicine and Dentistry, University of Alberta, Edmonton, AB Canada; 3 College of Biological and Food Engineering, Hubei Minzu University, Enshi, China; 4 Department of Biological Sciences, University of Alberta, Edmonton, AB Canada; 5 Department of Biochemistry, University of Alberta, Edmonton, AB Canada

**Keywords:** Polycystic kidney disease, Kidney

## Abstract

Autosomal dominant polycystic kidney disease (ADPKD) is one of the most common monogenic genetic disorders, caused by mutations in receptor PKD1 or ion channel PKD2, and is characterized by progressive renal cyst development with additional hepatic and extrarenal manifestations. As effective treatments for ADPKD remain limited, further investigation into the function and regulation of PKD proteins is needed. Using biotin-based proximity labeling combined with mass spectrometry in human embryonic kidney (HEK) cells, here we identify Ras association domain family member 4 (RASSF4) as a potential PKD2-interacting protein. The association between PKD2 and RASSF4 is validated by co-immunoprecipitation, bimolecular fluorescence complementation, and in vitro binding assays in HEK cells and mouse kidneys. Functional analyses using two-electrode voltage clamp electrophysiology in *Xenopus* oocytes demonstrate that RASSF4 enhances PKD2 channel activity without affecting its membrane expression. In vivo studies in larval zebrafish show that RASSF4 over-expression alleviates, whereas Rassf4 knockdown exacerbates, Pkd2 knockdown–associated phenotypes, including tail curling, pronephric cyst formation, renal filtration defects, and motor dysfunction. Disruption of the RASSF4/PKD2 interaction using a blocking peptide (amino acid P134–S168) abolishes RASSF4-mediated stimulation of PKD2 channel function and phenotypic rescue, while worsening disease severity, likely by interfering with endogenous complex formation. Mechanistically, RASSF4 enhances the functionally critical intramolecular interaction between the PKD2 N- and C-termini and suppresses RAS/MAPK signaling in HEK cells. Together, these findings identify RASSF4 as a PKD2 regulator and suggest that the RASSF4/PKD2 complex represents a potential therapeutic target for ADPKD.

## Abstract (web summary)

RASSF4 associates with PKD2 to enhance its channel function and suppress RAS/MAPK signaling, mitigating polycystic kidney disease phenotypes and highlighting a potential therapeutic target for ADPKD.

## Introduction

Autosomal dominant polycystic kidney disease (ADPKD) is an inherited kidney disease characterized by the formation of multiple cysts in the kidneys, which can eventually lead to kidney failure. ADPKD is one of the most common genetic kidney diseases, with an incidence of approximately 1:1000 individuals^1,2^. The main pathogenic genes responsible for ADPKD are *PKD1* and *PKD2*, located on chromosomes 16 and 4, respectively. Mutations in *PKD1* cause approximately 80% of the ADPKD cases, while PKD2 mutations typically lead to milder symptoms, accounting for about 15% of ADPKD^3^. The function of PKD1 or PKD2 is regulated by a number of signaling factors or pathways, such as Ca^2+^, cAMP, mammalian target of rapamycin (mTOR) mTOR, WNT, vascular endothelial growth factor, Hippo, and RAS/MAPK^4–6^. Altered protein expression of PKD1 or PKD2 also leads to cystic phenotypes in animal models^7,8^, indicating that dosage matters.

PKD2 is a member of the transient receptor potential (TRP) superfamily of cation channels and is also named TRP polycystin-2 (TRPP1) according to the IUPHAR/BPS guide to pharmacology (https://www.guidetopharmacology.org/). All the TRP members share similar membrane topology, with six transmembrane segments (S1 to S6), a pore loop between S5 and S6, and N- and C-termini located intracellularly^4,9,10^. Interestingly, PKD2 possesses a large (>200 amino acid residues) extracellular S1–S2 loop that contains at least 15 pathogenic missense mutations and modulates channel assembly^9,11^. The loop may contain binding sites for extracellular ligands and regulate channel gating^12^. PKD2 is expressed in various tissues, with high expression in the kidney, particularly in the nephron epithelial cells^13^.

PKD2 interacts with PKD1, forming a 1:3 heterotetrametric PKD1/PKD2 channel complex that is crucial for calcium signaling and cell proliferation^11,14–16^. PKD2 interacts with other TRP channels, including TRPV4, TRPC1, and TRPC5, forming channel complexes with unique biophysical properties^17–19^ and a number of other proteins, such as IP3 receptor, TACAN, eIF2α, and cytoskeleton proteins^20–24^, through which it exercises various functional roles.

Ras-association domain family member 4 (RASSF4) is one of the 10 members in the family and contains C-terminal Ras association (RA) and Salvador RASSF-Hippo (SARAH) domains^25–28^. RASSF4 is widely expressed, and its expression is down-regulated in human tumor cells due to promoter methylation, making it a tumor-suppressor^29,30^. RASSF4 is a scaffold protein that interacts with a variety of other proteins to regulate multiple cellular processes, such as cell cycle progression, apoptosis, cell migration, and response to DNA damage^28,31^. The functional significance of RASSF4 lies in its ability to coordinate signaling networks through interaction with key partner proteins, such as those in Ras and Hippo signaling-pathways^29,32,33^.

Using PKD2 for proximity labeling and mass spectrometry analysis, we identified RASSF4, a known regulator of RAS/MAPK signaling, as one of the potential interacting partners of PKD2. This study examines how RASSF4 physically and functionally interacts with PKD2 in *Xenopus* oocytes and human embryonic kidney (HEK293T) cells and how it modulates PKD2-associated cystic disorders in zebrafish. Experiments were undertaken using two-electrode voltage clamp electrophysiology with oocytes to characterize regulation of the PKD2 channel function by RASSF4, and a blocking peptide strategy was used to determine the importance of the RASSF4/PKD2 binding for PKD2 intramolecular interactions, channel function, cell cycle progression, PKD2-dependent disease development in larval zebrafish, and activity of RAS/MAPK signaling.

## Results

### Physical interaction between PKD2 and RASSF4

**Fig. 1:**
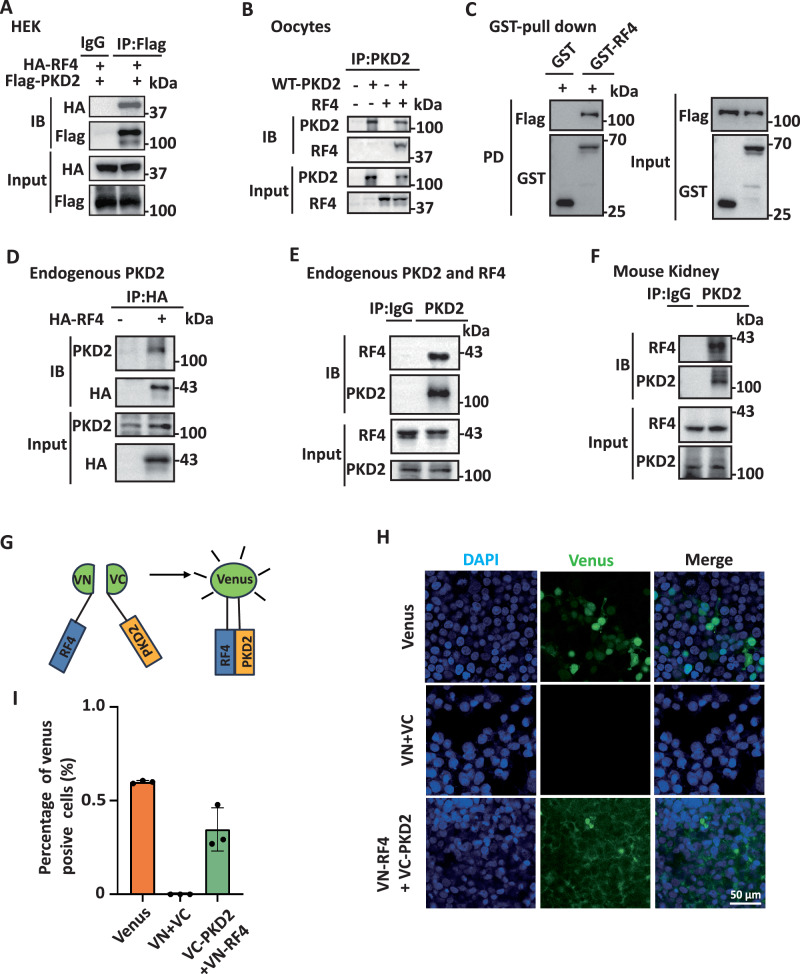
Physical interaction between PKD2 and RASSF4. Representative data showing interaction between exogenous RASSF4 (RF4) and PKD2 in HEK cells (**A**) and *Xenopus* oocytes (**B**) detected using co-IP assays and WB. **C** Representative data showing interaction between purified GST-tagged RASSF4, expressed in *E. coli* BL21, and Flag-tagged PKD2, expressed in HEK293T cells, assessed by GST pull-down assays. Representative data showing interaction of over-expressed (**D**) and endogenous (**E**) RASSF4 with endogenous PKD2 in HEK cells, examined using co-IP. **F** Representative data showing interaction between endogenous RASSF4 and PKD2 assessed by co-IP in a kidney of an 8-week-old C57BL/6 mouse. **G**–**I** The RASSF4/PKD2 interaction was analyzed using BiFC assays. **G** Schematic presentation of a BiFC assay. The Venus N-terminal half (VN) and C-terminal half (VC) were tagged to the N-terminal end of RASSF4 and PKD2, respectively, for transfection in HEK cells. The green fluorescent signal indicates proximity between VN and VC, which indicates the presence of the RASSF4/PKD2 association. **H** Representative data showing fluorescence in HEK cells transfected with Venus, VN + VC or VN-RF4 + VC-PKD2. Nuclei were stained with DAPI. **I** Statistical data showing quantified fluorescence intensities obtained under the same conditions as in (**H**).

When PKD2-containing complexes were enriched by biotin neighborhood labeling in HEK293T cells transiently overexpressing human PKD2 and subsequently analyzed by liquid chromatography-tandem mass spectrometry, several potential binding partner proteins, including RASSF4, were generated (Fig. S1). RASSF4 was then selected for further analyses in the present study^34,35^. The interaction between PKD2 and RASSF4 was verified by co-immunoprecipitation (co-IP) analysis in transfected HEK cells and oocytes (Fig. 1A, B). GST pull-down assays using recombinant GST-RASSF4 and Flag-PKD2 expressed in HEK cells confirmed the RASSF4/PKD2 interaction (Fig. 1C). The RASSF4/PKD2 complexing was further documented by co-IP assays when one or both proteins were endogenously expressed and by the bimolecular fluorescence complementation (BiFC) analysis (Fig. 1D–I). These findings together firmly showed that RASSF4 is a novel interacting partner of PKD2.

### Effects of RASSF4 on the PKD2 channel function

**Fig. 2:**
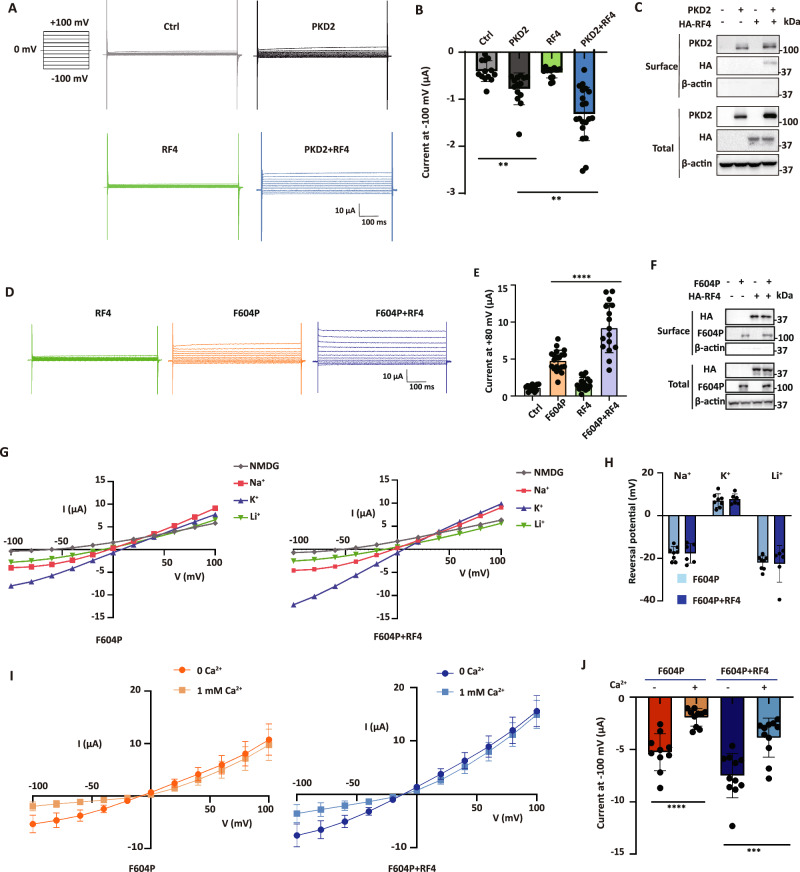
Effect of RASSF4 on PKD2 channel function in *Xenopus* oocytes. **A** Oocytes were co-injected with antisense thiophosphate oligonucleotides targeting connexin 38 (aCnx38) to minimize the endogenous currents induced in the presence of divalent cation-free extracellular solutions, similarly as described^64^. TEVC experiments were carried out in the extracellular Na^+^-containing divalent cation-free solution (in mM: 100 NaCl, 2 KCl, 10 Hepes, pH 7.5). A voltage-jump protocol (left panel) was used to generate representative current traces (right panels) obtained in oocytes overexpressing RASSF4, PKD2, PKD2 + RASSF4 or none (Ctrl). **B** Averaged steady-state currents at −100 mV under the same experimental conditions as in (**A**). Data are presented as the mean ± SD, *n* = 13–20 oocytes for each condition. **p < 0.01, by unpaired Student’s *t* test. Exact *p* values: Ctrl vs. PKD2, *p* = 0.0024; PKD2 vs PKD2 + RF4, *p* = 0.0035. **C** Representative biotinylation data showing the biotinylated (surface) and total protein expression of PKD2 and RASSF4. **D** Representative current traces showing the effect of RASSF4 on the function of PKD2 mutant F604P, under similar experimental conditions as in (**A**), except that 0.3 mM MgCl₂ was added to the extracellular solution and that oocytes were not injected with aCnx38. *n* = 15–20 oocytes for each condition. **E** Averaged steady-state currents at +80 mV under the same experimental conditions as in (**D**). Data are presented as the mean ± SD, with *n* = 15–20 oocytes. ****p < 0.0001, by unpaired Student’s *t* test. **F** Representative biotinylation data showing the biotinylated (surface) and total expression of PKD2-F604P and RASSF4. **G** Effect of RASSF4 on the cation selectivity of mutant F604P. Averaged *I*–*V* curves were obtained in the presence of different monovalent cations, as indicated, under the same experimental conditions as in (**A**) (*n* = 8 and 6 oocytes for F604P and F604P + RASSF4, respectively) and have been subtracted by the averaged *I*–*V* curves from control oocytes (*n* = 7). The Na^+^-containing solution was replaced by equimolar K^+^, Li^+^, or NMDG. Data are presented as the mean ± SD. **H** Averaged reversal potentials obtained from data in (**G**). Data are presented as the mean ± SD, with *n* = 6–8 oocytes. Averaged *I*–*V* curves (**I**) and currents at −100 mV (**J**) obtained under similar conditions as in (**G**) with or without 1 mM Ca^2+^ (*n* = 10 and 11 oocytes for F604P and F604P + RASSF4, respectively), with subtraction by averaged currents obtained from control oocytes (*n* = 10). Data are presented as the mean ± SD. ****p < 0.0001 and ***p < 0.001, by paired Student’s *t* test for the effect of Ca^2+^. Exact *p* value: F604P + RF4, with Ca^2+^ vs no Ca^2+^, *p* = 0.0004.

To investigate the functional role of RASSF4 in PKD2, we performed electrophysiological analyses using *Xenopus* oocytes as an expression model. While the basal channel activity of wild-type (WT) human PKD2 was barely detectable, consistent with previous reports^24,36^, co-expression of RASSF4 significantly enhanced the PKD2 channel function (Fig. 2A, B), given that biotinylation assays revealed no significant effect of RASSF4 on the PKD2 surface expression in oocytes as well as in HEK cells (Figs. 2C and S2). We next employed the PKD2 activated mutant F604P^36^and found that RASSF4 also significantly increases its channel function of and associates with the mutant in oocytes (Fig. 2D–F). Our data together demonstrated that RASSF4 substantially stimulates the PKD2 channel function.

We also investigated whether RASSF4 affects the ion selectivity of F604P using extracellular solutions containing 100 mM Na^+^, Li^+^, K^+^, or N-methyl-D-glucamine (NMDG, as a control). We found that the reversal potentials obtained in the presence of different solutions are not significantly affected by RASSF4 co-expression (Fig. 2G, H), indicating that RASSF4 did not significantly affect the cation selectivity. Further, it is known that extracellular Ca^2+^ reduces the conductance of PKD2 to monovalent cations^36^. Here, we found that RASSF4 does not significantly affect the inhibitory effect of Ca^2+^ on mutant F604P (Fig. 2I, J). In summary, our data demonstrate that RASSF4 promotes the activity of the PKD2 channel without significantly affecting its surface membrane expression, cation selectivity or the inhibitory effect of Ca^2+^.

### Regulation of Pkd2-associated disease phenotypes by RASSF4 in zebrafish

**Fig. 3:**
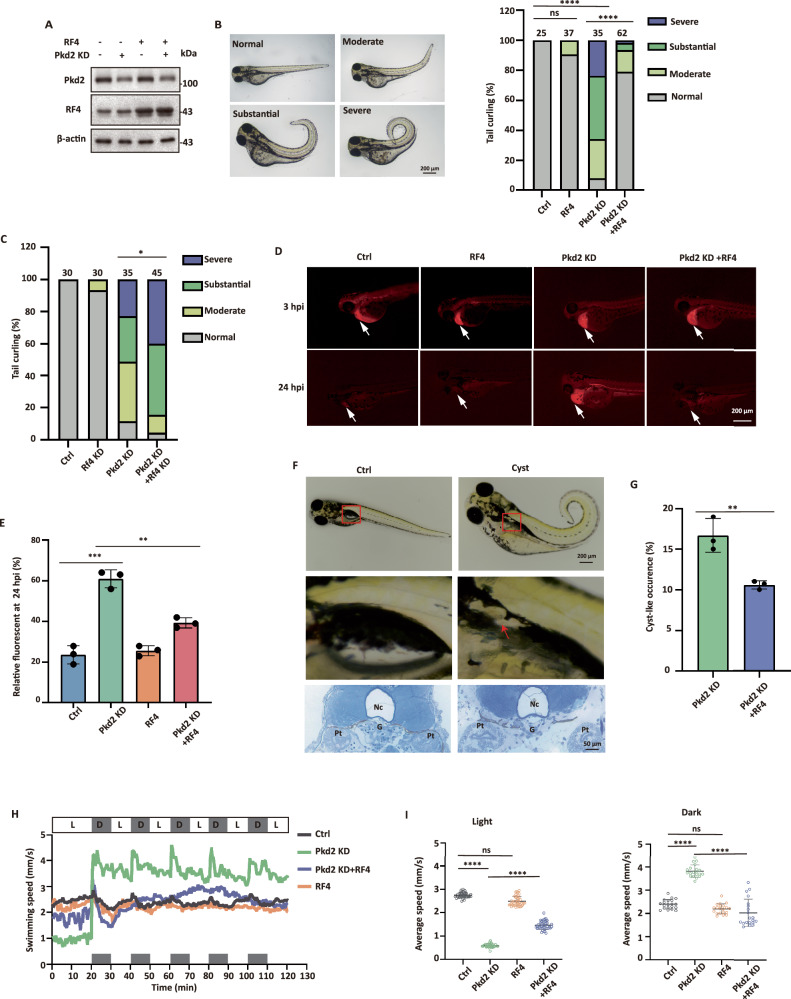
Effects of RASSF4 on tail curling, pronephric filtration, pronephric cystogenesis, and light-dependent swimming defects in larval zebrafish with Pkd2 KD. **A** Representative WB data showing the expression of Pkd2 and Rassf4 in 5-dpf zebrafish. **B** Effect of human RASSF OE on zebrafish tail curling induced by Pkd2 KD. Left panel, representative pictures presenting different severity levels of tail curling: normal (no curvature), moderate (0° < curvature angle < 90°), substantial (90°–180°), and severe (>180°). Right panel, distribution of the four tail curling severities under the indicated experimental conditions, with “Ctrl” indicating water injection. The total number of zebrafish used under each condition, from three independent experiments, is indicated. *pkd2* KD: micro-injection of 200 pg *pkd2*-targeting sgRNA + 300 pg Cas9 mRNA; *rassf4* OE: micro-injection of 100 pg *rassf4* mRNA. ****p < 0.0001, and ns, not significant, by chi-square test. **C** Effect of Rassf4 KD on the severity distribution of tail curvature induced by Pkd2 KD. *p < 0.05, by chi-square test. **D** Effect of RASSF4 OE on the renal filtration defect induced by Pkd2 KD. Larval zebrafish at 3 dpf were injected with rhodamine dextran dye into the pericardial cavity. Representative fluorescence intensity measured at 3 and 24 h post-injection (hpi). Arrows indicate the heart. **E** Averaged residual fluorescence intensity at 24 hpi (relative to the value at 3 hpi). Data are presented as the mean ± SD, with *n* = 6–9. **p < 0.01, and ***p < 0.001, by unpaired Student’s *t* test. **F** Representative images of whole body and histological sections of 5-dpf larval zebrafish, showing the presence of cyst-like pronephric abnormalities, including tubule dilation and fluid around the tubule in fish with Pkd2 KD. G glomerulus, Pt pronephric tubule, Nc notochord. **G** Averaged cyst-like occurrence % obtained in 5-dpf zebrafish under indicated conditions, with cyst-like abnormalities identified as in (**F**), from three independent experiments. Cyst-like occurrence % was calculated from *n* = 20 to 28 fish for each condition in each experiment. Data are presented as the mean ± SD. **p < 0.01, by unpaired Student’s *t* test. **H** Representative swimming speed of 5-dpf larvae recorded at 30 s/sample under either the light (L) or dark (D) condition, each for 10 min. **I** Averaged swimming speed of 5-dpf larval zebrafish with or without Pkd2 KD and RASSF4 OE under the initial light for 90 min (left panel) and first dark (right panel) conditions. Data are presented as the mean ± SD, with *n* = 48 for each condition. ****p < 0.0001, by one-way ANOVA analysis.

Loss or reduction of Pkd2 in larval zebrafish results in the development of curved tail and pronephric cysts resembling renal cysts in mammals^37,38^. Because RASSF4 enhanced the Pkd2 channel function in oocytes, we wondered whether it has a similar effect on Pkd2 in zebrafish, through which it demonstrates a rescuing effect. For this, we employed the CRISPR/Cas9 method in zebrafish embryos to achieve *pkd2* knockdown (KD) and co-injected sgRNA targeting *pkd2* with Cas9 protein (Fig. S3A, B). We also employed RASSF4 over-expression (OE) via mRNA injection, with its protein expression shown in Fig. 3A. We found that in 5-day-post-fertilization (5 dpf) larval zebrafish with Pkd2 KD, RASSF4 OE significantly mitigated the tail curvature phenotype (Fig. 3B). Consistently, Rassf4 KD exacerbated the severity and occurrence of tail curvature associated with Pkd2 KD (Figs. 3C and S3C, D), while Rassf4 KD alone did not induce noticeable pathological phenotype (Fig. 3C), suggesting that Pkd2 function in the fish was not low enough to induce the associated diseases. Furthermore, Pkd2 KD resulted in defective renal filtration assessed by rhodamine dextran clearance assays, which was alleviated by RASSF4 OE (Fig. 3D). Pkd2 KD also induced pronephros cyst formation, which was markedly reduced by RASSF4 OE (Fig. 3E–G). In Pkd2 KD larval zebrafish exhibiting cystic pronephros and a curled tail, locomotor analysis revealed significantly reduced baseline swimming speed in the presence of light compared to fish with WT Pkd2. Interestingly, only fish with Pkd2 KD exhibited a substantially increased swimming speed upon dark stimulus and repeated dark/light stimuli, each for 10 minutes (min) (Fig. 3H, I). Consistently, co-expression of Rassf4 largely rescued the Pkd2 KD-associated anomalies, presumably through enhancing the Pkd2 function. These results together indicate that Rassf4 enhances the Pkd2 function in vivo in larval zebrafish with Pkd2 KD, through which it ameliorates the tail curvature, glomerular filtration function, pronephric cyst formation, and light-dependent swimming speed defects. Collectively, our data revealed that Rassf4 partially rescues Pkd2-deficiency- induced developmental abnormalities and pronephric dysfunction in zebrafish.

### Domains mediating the PKD2/RASSF4 interaction

**Fig. 4:**
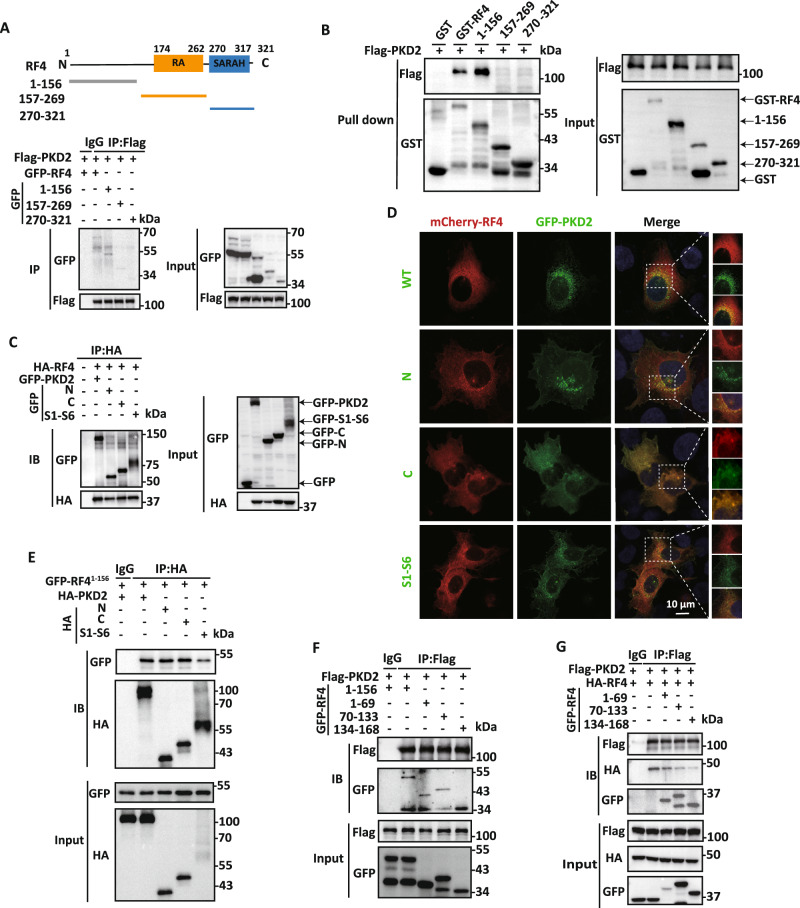
Domains mediating the RASSF4/PKD2 interaction. **A** Upper panel, schematic architecture of human RASSF4 protein, with key functional domains indicated. Lower panels, representative co-IP data from HEK cells, showing interaction of Flag-PKD2 with GFP-RASSF4 and three GFP-tagged RASSF4 fragments. **B** Representative data obtained from GST pull-down assays showing the RASSF4/PKD2 interaction. HEK cells expressing Flag-PKD2 were incubated with purified GST-RASSF4 truncation proteins. Complexes were pulled down with glutathione beads and analyzed by WB. **C** Representative data from co-IP assays in HEK cells co-expressing HA-RASSF4 with different GFP-tagged PKD2 fragments (left panels) or co-expressing GFP-RASSF4 with different HA-tagged PKD2 fragments (right panels). “N”, PKD2 N-terminus (M1-S219), “C”, C-terminus (I671-V968), and “S1–S6”, central domain (K215-M675). **D** Representative confocal microscopy images showing subcellular colocalization in U-2 OS cells of mCherry-RASSF4 with different GFP-PKD2 constructs, as indicated. Scale bar: 10 μm. **E** Representative co-IP data showing interaction of GFP-tagged RASSF4-M1-P156 with different HA-PKD2 fragments, as indicated, in HEK cells. **F**, **G** Representative co-IP data showing interaction of Flag-PKD2 with different GFP-tagged RASSF4 fragments, as indicated, in HEK cells.

We next wanted to determine the domains in RASSF4 and PKD2 that mediate their association. For this, we first divided RASSF4 into three domains, N-terminal M1-P156, RA domain G157-E269, and SARAH domain plus the C-terminus V270-K321, and found by co-IP assays in HEK cells that PKD2 interacts with the M1-P156 fragment (Fig. 4A), which was confirmed by GST pull-down assays (Fig. 4B). We next divided PKD2 into three domains, the N-terminus M1-S219, the central domain containing S1 to S6 (S1–S6), and the C-terminus I671-V968. Interestingly, co-IP assays in HEK cells demonstrated that RASSF4 interacts with all these three PKD2 domains (Fig. 4C), which was supported by our colocalization data obtained using confocal microscopy (Fig. 4D). Further, we found that the RASSF4 fragment M1-P156 is sufficient to associate with each of the three PKD2 domains (Fig. 4E). This suggests the possibility that this N-terminal region of RASSF4 contains multiple domains that interact with PKD2. To test this possibility, we transfected plasmids encoding the RASSF4 fragments M1-L69, Q70-A133, and P134-S168, and indeed found that all of them interact with PKD2 in HEK cells, with strongest P134-S168/PKD2 binding (Fig. 4F). We then co-expressed each of the RASSF4 fragments M1-L69, Q70-A133, and P134-S168 which act as potential blocking peptides with full-length RASSF4 and PKD2, expecting that one or more of these fragments will disrupt the RASSF4/PKD2 complexing through competitive binding. Indeed, we found that P134-S168 significantly blocks (reduces) the RASSF4/PKD2 complexing assessed by co-IP (Fig. 4G). These data together suggest the presence of at least three binding contacts between RASSF4 and PKD2, presumably mediated by M1-L69, Q70-A133 and P134-S168 of RASSF4, paired with the three PKD2 domains (Fig. 4C–E).

### Requirement of the RASSF4/PKD2 association for regulation of the PKD2 channel function and PKD2-dependent disease phenotypes by RASSF4

**Fig. 5:**
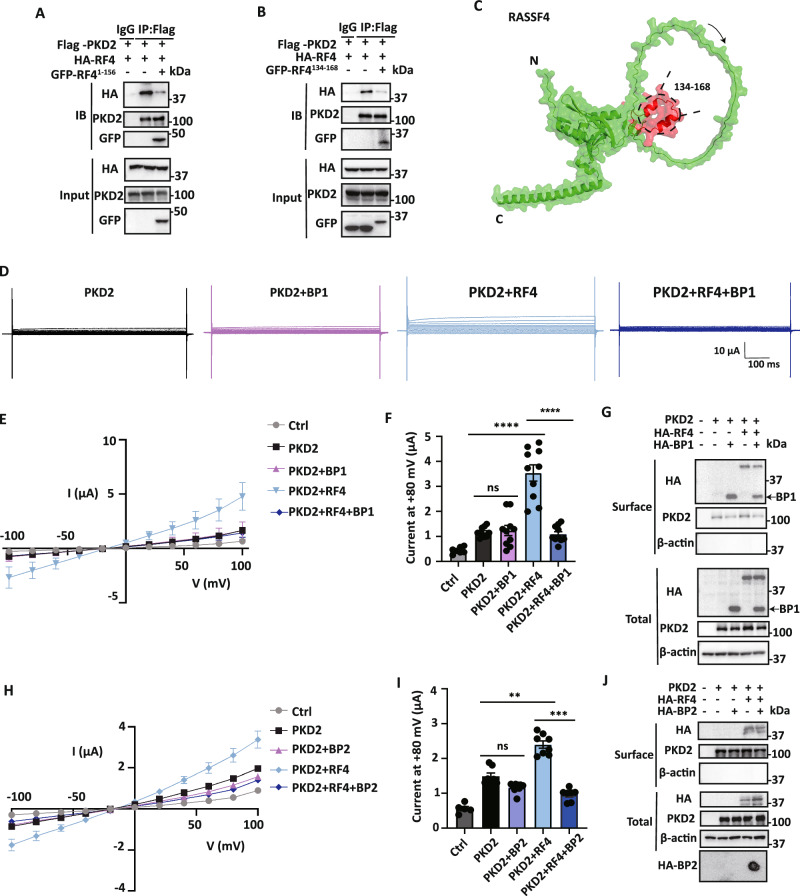
Effects of blocking peptides on the RASSF4/PKD2 binding and functional regulation of PKD2 by RASSF4. The blocking effect of fragments M1-P156 (BP1) (**A**) and P134-S168 (BP2) (**B**) on the RASSF4/PKD2 interaction was shown by representative co-IP data obtained from HEK cells. **C** The Alphafold3 program predicted the presence of PKD2-binding, α-helical fragment in the RASSF4 N-terminus (P134-S168). **D** With similar electrophysiology conditions as in Fig. 1A, representative current traces obtained from oocytes expressing different constructs, as indicated, showed the blocking effect of BP1. **E**, **F** Averaged *I*–*V* curves and currents at +80 mV obtained under the same conditions as in (**D**), showing the blocking effect of BP1. Data are presented as the mean ±  SD, with *n* = 7–10 oocytes for each condition. ****p < 0.0001, ns, not significant, by unpaired Student’s *t* test. **G** Representative biotinylation data showing the effect of HA-BP1 on the surface expression of PKD2 and RASSF4. **H**, **I** Averaged *I*–*V* curves and currents at + 80 mV obtained under similar conditions as in (**D**), showing the blocking effect of HA-BP2. *n* = 5–10 oocytes (**H**) and *n* = 5–10 oocytes (**I**) for each condition. Data are presented as mean ± SD. **p < 0.01, ***p < 0.001; ns, not significant, by unpaired Student’s *t* test. **J** Representative biotinylation data showing the effect of HA-BP2 on the surface expression of PKD2 and RASSF4. For dot blot analysis of HA-BP2, samples (2 µL) prepared by biotin labeling were spotted on a methanol-pretreated PVDF membrane, dried and UV-crosslinked. After blocking with 5% milk, BP2 was detected using an HA antibody. All subsequent steps were performed according to our WB protocol.

We next wanted to examine whether the RASSF4/PKD2 interaction is important for RASSF4 to regulate the PKD2 function, using a blocking peptide strategy. A peptide that disrupts or significantly reduces the RASSF4/PKD2 binding is called a blocking peptide. We then tested M1-P156 and P134-S168 for their potential as blocking peptides by co-IP assays and indeed found that they are able to substantially disrupt the RASSF4/PKD2 association (Fig. 5A, B) and thus are named blocking peptides 1 and 2 (BP1 and BP2), respectively. Interestingly, analysis using the AlphaFold3 program predicted that the RASSF4 domain (P134-S168) contains two α-helices (Fig. 5C). The structure of the α-helices and the specific amino acid residues provide favorable conditions for protein-protein interactions. Next, we evaluated the effect of each blocking peptide on the stimulation of the PKD2 channel function by RASSF4 in oocytes and found that each of BP1 and BP2 abolishes the functional stimulation of the PKD2 channel by RASSF4, while themselves did not significantly affect the PKD2’s function or the expression of PKD2 and RASSF4 (Fig. 5D–J). These data together demonstrate that the RASSF4/PKD2 binding is required for the functional regulation of PKD2 by RASSF4.

**Fig. 6:**
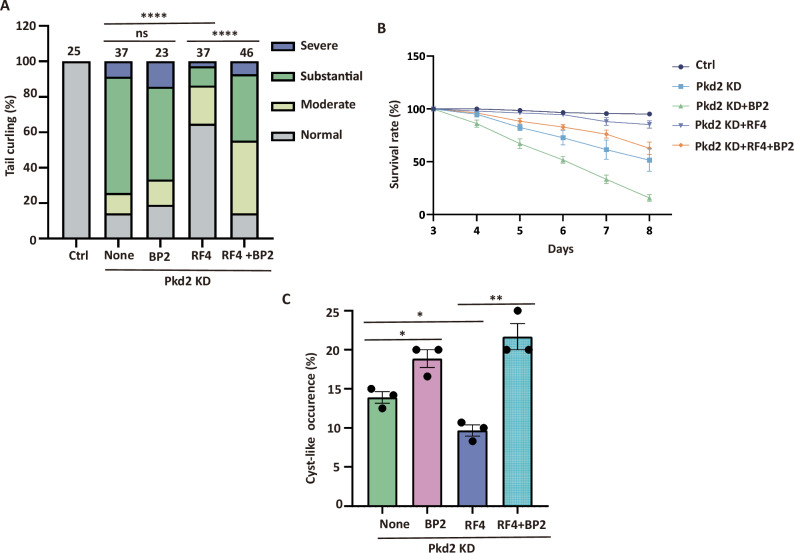
Effect of blocking peptide BP2 on Pkd2-dependent disease phenotypes in larval zebrafish. **A** Similar experimental conditions as in Fig. 3C. Distribution of the four tail curling severities under four different experimental conditions: with or without Pkd2 KD, RASSF4 OE, and BP2 OE, as indicated, with “Ctrl” indicating water injection. The total number of zebrafish used under each condition, from three independent experiments, is indicated. BP2 OE: micro-injection of 200 pg BP2 mRNA. ****p < 0.0001, by chi-square test. **B** Survival rates of different groups of larval zebrafish over an 8-day period. **C** Similarly as in Fig. 3F, the averaged cyst-like occurrence % obtained in 5-dpf zebrafish under the indicated conditions from three independent experiments, with 15–30 fish used for calculating the cyst-like occurrence % for each condition in each experiment. Data are presented as the mean ± SD. *p < 0.05, **p < 0.01, by unpaired Student’s *t* test.

We also examined the importance of the physical RASSF4/PKD2 interaction for the regulation of PKD2-dependent disease phenotypes in larval zebrafish by RASSF4. While RASSF4 substantially ameliorated the disease phenotypes in zebrafish with Pkd2 KD, including tail curling severity and prevalence, survival rate, and pronephric cyst occurrence, expression of blocking peptide BP2 significantly reduced the effect of RASSF4 (Fig. 6), consistent with the blocking effect of BP2 in oocytes. Additionally, when BP2 is over-expressed in zebrafish with Pkd2 KD, these phenotypes significantly worsened, suggesting disruption of the endogenous RASSF4/PKD2 complexing by BP2. Therefore, these data together strongly support the conclusion made using oocytes and electrophysiology that the physical RASSF4/PKD2 binding mediates the functional regulation of PKD2 by RASSF4.

### Effects of the RASSF4/PKD2 association on the PKD2 intramolecular interaction, activity of the RAS/MAPK signaling, and cell cycle progression

**Fig. 7:**
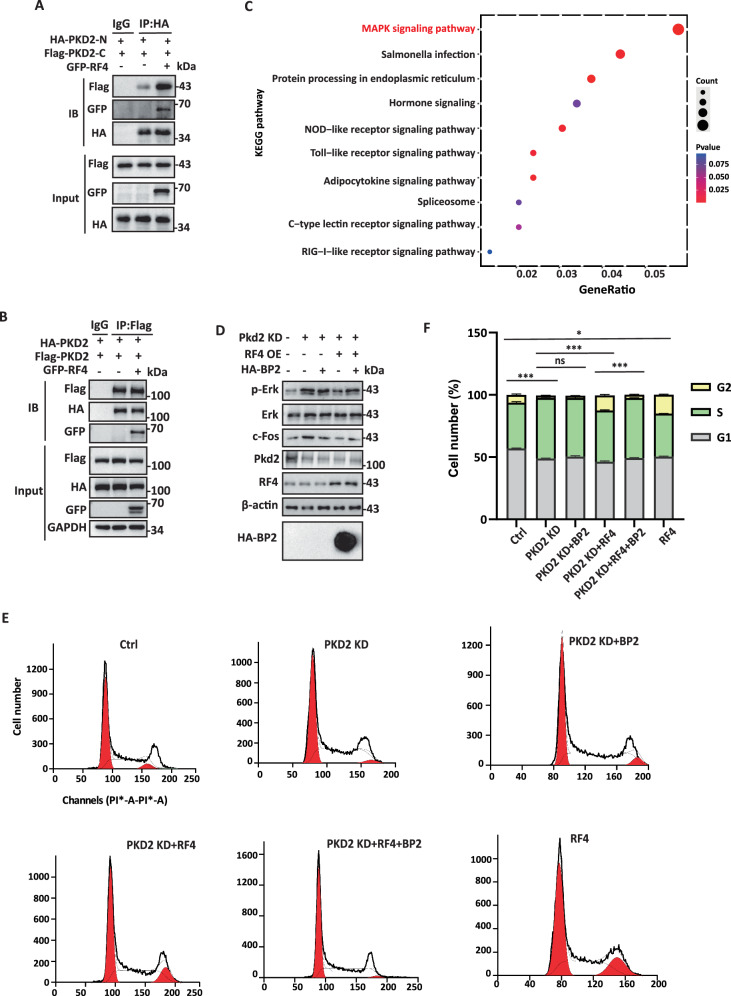
Effects of RASSF4 or BP2 on the PKD2 N/C binding, RAS/MAPK signaling pathway activity, and cell cycle progression. **A** Representative co-IP data using HEK cells, showing the effect of RASSF4 OE on the intramolecular N/C binding assessed by co-IP strength between PKD2-N (M1-S219) and PKD2-C (I671-V968). **B** Representative co-IP data using HEK cells, showing the effect of RASSF4 on the PKD2 intermolecular binding assessed by co-IP strength between HA-PKD2 and Flag-PKD2. **C** KEGG pathway enrichment (transcriptome) analysis (by means of RNA-seq assays and the Hypergeometric test) in 5-dpf zebrafish was performed for calculating the ratio of the number of significantly upregulated genes under the PKD2 KD to that under the WT PKD2 condition. The dot size indicates the number of upregulated genes (count), and the dot color represents the *p*-value to indicate the significance of the enrichment. Fifty zebrafish larvae were collected for each assay condition, and shown result was averaged from three independent experiments. **D** Representative WB data showing the effect of HA-BP2 OE on the ERK and p-ERK levels in 5-dpf zebrafish larvae with PKD2 KD and RASSF4 OE. **E** Representative cell cycle data obtained using transfected MDCK cells with PKD2 KD by shRNA, HA-RASSF4 OE, or HA-BP2 OE. Cells at 80% confluency were used for transient transfections, and 36 h after were harvested for propidium iodide (PI) staining and flow cytometry analysis. PI fluorescence intensity, which reflects the DNA content, was detected using flow cytometry. The number of detected cells (y-axis) was plotted as a function of the number of PI fluorescence intensity (x-axis) under different conditions, as indicated. **F** Statistical data obtained as in (**E**), showing cell cycle distribution averaged from three independent experiments, presented as the mean ± SD. ***p < 0.001, and ***p < 0.001, ns, not significant, by one-way ANOVA analysis.

While we have shown the requirement of the physical RASSF4/PKD2 binding for the functional regulation of PKD2 by RASSF4, we asked how the binding of RASSF4 to PKD2 enhances its function. We previously reported that the intramolecular binding between the N- and C-terminus (the so-called N/C binding) is essential for the channel function of several TRPs, including PKD2^39^. The channel activity of GOF PKD2 mutant F604P was completely abolished by point mutations that disrupt the N/C binding and was substantially reduced by a blocking peptide that largely disrupts the N/C binding^39^. Here, we found that RASSF4 expression significantly increases the N/C binding strength (Fig. 7A) but does not affect the binding between two full-length PKD2 (Fig. 7B), suggesting the possibility that RASSF4 enhances the PKD2 intramolecular N/C binding through the RASSF4/PKD2 interaction, thereby stimulating its channel function.

Because the RAS/MAPK signaling pathway is up-regulated in cystic cells^40^, we investigated how the RASSF4/PKD2 interaction regulates this pathway in zebrafish larvae with WT Pkd2 or Pkd2 KD. We first confirmed by transcriptomic KEGG enrichment analysis that Pkd2 KD indeed induced significant alterations in signaling pathways, including upregulation of the RAS/MAPK pathway (Fig. 7C). We assessed the RAS/MAPK pathway activity by detecting the phosphorylation level of its downstream molecule ERK with Western blotting (WB). While ERK phosphorylation increased significantly in the presence of Pkd2 KD, it was significantly suppressed by RASSF4 OE, and this suppression was abolished by co-expression of BP2 (Figs. 7D and S4). Because BP2 OE without RASSF4 OE (Fig. 7D) or RASSF4 OE alone (Fig. S4) had no significant effect on ERK phosphorylation, these data indicated that the RASSF4/PKD2 association is important for activation of the RAS/MAPK pathway.

Cystic cells are known to have increased population in the S-phase and increased cell proliferation^41,42^. We indeed confirmed by flow cytometry (by propidium iodide staining) that Madin–Darby canine kidney cells (MDCK) cells with PKD2 KD by shRNA exhibit much increased population of S-phase cells (and reduced G2/M-phase cells) (Fig. 7E). RASSF4 OE suppressed the increase and this suppression was abolished by BP2 OE while BP2 in the absence of RASSF4 OE had no significant effect (Fig. 7F), indicating the importance of the RASSF4/PKD2 interaction for the cell cycle regulation. We noticed that RASSF4 OE alone significantly decreases the S phase (Fig. 7F), which is consistent with its role as a tumor suppressor.

**Fig. 8:**
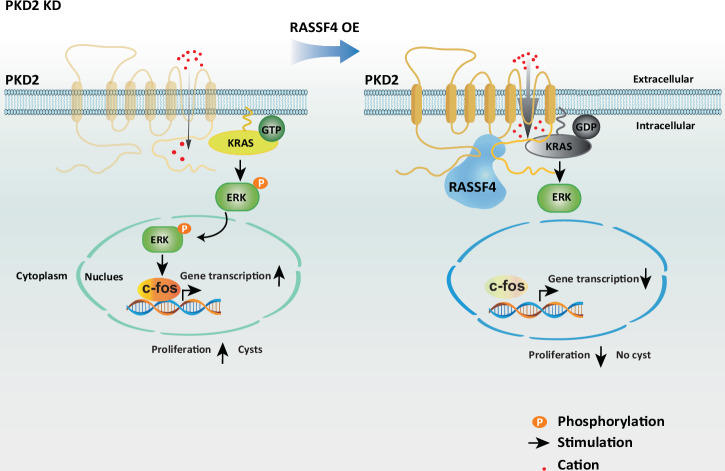
Proposed model for RASSF4-mediated regulation of PKD2 function and downstream signaling. Left panel, PKD2 KD reduces PKD2 channel function, resulting in aberrant activation of the RAS/MAPK signaling pathway, excessive cell proliferation, and cyst progression. Right panel, RASSF4 overexpression promotes PKD2 intramolecular N/C binding, enhances PKD2 channel function, and suppresses RAS/MAPK signaling, thereby alleviating PKD2 KD-associated over-proliferation and cyst formation.

These results together support the conclusion that the RASSF4/PKD2 interaction is important for the inhibition of cell over-proliferation associated with ADPKD, presumably through the RAS/MAPK pathway and through the PKD2 intramolecular N/C binding and channel function (see working model, Fig. 8).

## Discussion

Our present study discovered that RASSF4 up-regulates the PKD2 function through direct physical binding and ameliorates Pkd2-dependent disease phenotypes in larval zebrafish via the RAS/MAPK pathway. The Ras-association domain of RASSF4 mediates interaction with small G proteins, which regulates proliferation, migration^29,31,32^, and the SARAH domain mediates auto-dimerization and interaction with other proteins, such as core components of the Hippo pathway, which regulates apoptosis and tissue growth^27,43,44^. In contrast, the functional role of the RASSF4 N-terminus remains unknown. Our present study found that RASSF4 binds with PKD2 only through RASSF4-M1-P156. Interestingly, while three PKD2 domains (N-terminus, central domain and C-terminus) interact with RASSF4-M1-P156, we indeed identified three N-terminal fragments of RASSF4 interact with PKD2, suggesting the presence of three bonds between the RASSF4 N-terminus and PKD2, as depicted in our working model (Fig. 8). Consistent with the reported physical proximity or interaction among the pre-S1, TRP domain and S4-S5 linker of TRP channels^12,39,45^, in the model, we think that each of the three domains binds with the RASSF4 N-terminus, which enhances the N/C binding and channel function, thereby suppressing proliferation and alleviating cystic disorders via the RAS/MAPK/ERK pathway. Finally, because PKD1 and PKD2 form a channel complex^15,16^, it remains to be determined whether and how RASSF4 regulates the PKD1/PKD2 complexing.

In this study, we characterized the physical interaction between RASSF4 and PKD2 and identified domains in both proteins that mediate the interaction in *Xenopus* oocytes, mammalian cells and zebrafish by different techniques, including co-IP, GST pull-down, BiFC, and immunofluorescence. We also showed that RASSF4 promotes the PKD2 function assessed by PKD2-mediated electric currents in oocytes and PKD2 KD-associated disease phenotypes in larval zebrafish (including tail curling, pronephric cyst formation, swimming defects, and animal death) without affecting the PKD2 protein expression. However, these data do not automatically lead to the statement that RASSF4 promotes the PKD2 function through their physical association. To test this, one could examine whether mutations in RASSF4 or PKD2 that abolish or reduce the RASSF4/PKD2 association will inhibit the stimulation of RASSF4 on the PKD2 function. However, because such mutations may affect the normal function of RASSF4 or PKD2 itself, whether the inhibited stimulation is due to a disrupted RASSF4/PKD2 association remains unclear. To unambiguously determine the requirement of the physical RASSF4/PKD2 binding for functional inhibition, we employed a blocking peptide strategy in which both RASSF4 and PKD2 remained intact (WT). Specifically, we found that each of BP1 and BP2, as part of RASSF4-N (M1-P156 and P134-S168), respectively abolishes or substantially reduces the RASSF4/PKD2 association and the stimulatory effect of RASSF4 on the PKD2 channel function in oocytes, while itself has no significant functional effect on PKD2 (Fig. 5). Our data using zebrafish supported these in vitro data. CRISPR/Cas9- and morpholino-mediated KD of PKD2 in larval zebrafish nicely recapitulated the hallmark of polycystic kidney disease, as they result in pronephric cysts together with tail curling, also a specific phenotype at 5-dpf ^38,46,47^. Over-expression of BP2 significantly inhibited the rescuing effect of RASSF4 on the PKD2-dependent disease severity (Fig. 6). Thus, our in vitro and in vivo data together allowed making the conclusion that the physical RASSF4/PKD2 association is required for the functional stimulation of PKD2 by RASSF4.

While our study clearly showed that the RASSF4/PKD2 binding mediates the functional stimulation of PKD2 by RASSF4, one would wonder how the physical binding leads to PKD2 functional augmentation. We previously reported that the intramolecular N/C binding is critical for the channel function of PKD2 and its homologue PKD2L1^39^. The PKD2 activated mutant F604P became nonfunctional with disrupted N/C binding^10^, indicating that the channel activation induced by mutation F604P is mediated by the N/C binding. Indeed, RASSF4 binding to PKD2 increased the intramolecular N/C binding strength (Fig. 7A). Interestingly, although dimerization of PKD2 is an important part of forming a functional homotetrameric channel, RASSF4 did not alter intermolecular interaction between two PKD2 proteins (Fig. 7B). Whether and how RASSF4 binding affects functionally important conformations independent of the N/C binding remains to be determined.

The development and progression of ADPKD has been reported to be associated with activation of several downstream signaling pathways, particularly the RAS/MAPK pathway, that lead to cell over-proliferation, dedifferentiation and apoptosis^40,48,49^. Extracellular signal-regulated kinase inhibition slows disease progression in PKD2 mouse^40,49–51^. In fact, in larval zebrafish, we found that RASSF4 significantly represses the ERK phosphorylation through activation of the RAS/MAPK pathway by Pkd2 KD. Therefore, we envision that RASSF4 binds with PKD2 to enhance its N/C binding and function, thereby suppressing the RAS/MAPK signaling and therefore cell proliferation (Fig. 8).

This study found that zebrafish larvae with Pkd2 KD not only exhibit cystic pronephros mimicking mammalian polycystic kidney phenotype but also exhibited light-dependent swimming defects, representing a novel Pkd2-dependent phenotype likely reflecting defects in sensory or motor neurons, although the underlying mechanism remains unknown. It was recently reported that PKD2 forms a functional complex with the autophagy-related protein BECN1 in primary cilia of hypothalamic neuronal model N43/5 cells, in which the complex plays an important role in neuronal homeostasis and autophagosome formation^52^. Thus, the light-dependent swimming defects in Pkd2 KD zebrafish may be due to an abnormal cilium-autophagy axis or insufficient BECN1/PKD2 complexing in the hypothalamus or retinohypothalamic tract.

As a known tumor suppressor, RASSF4 expression is down-regulated during the malignant progression of multiple cancers^29,53^. Interestingly, ADPKD and cancers share several cellular characteristics, such as over-proliferation, dedifferentiation, and reduced apoptosis, as well as cellular signaling, such as RAS/MAPK and Wnt pathways^40,50,54^. Indeed, while PKD2 itself acts as an ADPKD suppressor, our current study fully supports a cyst suppressor role of RASSF4, through complexing with PKD2, enhancing its function, and together inhibiting cell growth via the RAS/MAPK pathway. Similarities between PKD2 and cancer may be used to help characterizing how RASSF4 is regulated in cystic cells, e.g., whether RASSF4 is down-regulated in PKD due to promoter methylation as in cancer^29^ and how it is involved in the shared signaling pathways, such as those related to RAS and mTOR^32,53^.

## Conclusion

Most recent clinical trials for ADPKD have been carried out to inhibit the growth of renal cysts rather than directly correcting the function of PKD1 or PKD2^55^. For example, Tolvaptan, as an approved drug, is a vasopressin receptor 2 antagonist that can slow down cyst and kidney volume growth through inhibiting renal water reabsorption, but has a limited efficacy, a lack of specificity, and significant side effects. Under physiological conditions, PKD1 and PKD2 are in complex with many other proteins. Understanding these interactions, which may be constitutive or dynamic, will constitute a basis for correcting their altered function due to pathogenic mutations or dysregulated expression. The mechanism of how RASSF4 physically interacts with and functionally regulates PKD2 in cellular and zebrafish models that have been revealed in our study, which can be presented as “RASSF4/PKD2 → N/C binding → PKD2 function --| p-ERK → over-proliferation and ADPKD”, may provide a new therapeutic avenue for PKD2-associated ADPKD.

## Materials and methods

### Cell lines, reagents, and antibodies

HEK293T and U-2 osteosarcoma (U-2 OS) cell lines were purchased from the cell center of the Institute of Biochemistry and Cell Biology, Chinese Academy of Sciences. Commercially purchased antibodies and their concentrations used in this study are described in Supplementary Table 1.

### Plasmid construction, cell culture, and transfection

The cDNAs encoding human RASSF4, human PKD2 and their mutants were amplified by PCR, verified by sequencing, and cloned into different vectors, including pcDNA3.0-3×HA (Miaolingbio, P9394), pcDNA3.1-RFP (HonorGene, HG-VPH0003), p3×Flag-CMV (Biofeng, E7908), pEGFP (Miaolingbio, P0134), pmCherry (Miaolingbio, P0475), and pGEX-4T1 (Addgene, 27458001). Human influenza haemagglutinin (HA)-tagged human PKD2 (HA-PKD2) in pGEMHE for *Xenopus* oocyte expression and PKD2 mutant F604P in pcDNA3.1 for mammalian cell expression were generous gifts of Dr. Yong Yu (St John’s University, New York, NY). HEK and U-2 OS cells were cultured in DMEM supplemented with L-glutamine, penicillin-streptomycin, and 10% fetal bovine serum at 37 °C in an incubator with humidified air and 5% CO_2_. Transfection of plasmids was performed using polyethylenimine (Sigma-Aldrich, 9002-98-6) according to the manufacturer’s protocols.

### *Xenopus* oocyte expression

The mRNAs encoding human PKD2, human RASSF4 and other proteins or protein fragments were synthesized by in vitro transcription using the T7 mMESSAGE mMACHINE kit (Thermo Fisher, AM1344) and injected into oocytes (25 ng each) as described previously^10^. Control oocytes were injected with the same volume of water. Oocytes were incubated at 18 °C for 2–3 days before measurements.

### Co-immunoprecipitation

Co-IP experiments were performed based on previously described^56,57^. Briefly, mammalian cells and oocytes were washed with phosphate-buffered saline (PBS) and solubilized in ice-cold CelLytic M lysis buffer supplemented with a proteinase inhibitor cocktail. Supernatants were collected after centrifugation at 13,200 rpm for 20 min and pre-cleaned with 30% protein A/G magic beads (Thermo Fisher, 88802) for 1–2 h, followed by incubation with an indicated antibody at 4 °C for 5 h. After the addition of 25 μL of 30% protein A/G magic beads, the mixture was incubated overnight at 4 °C with gentle shaking. Immunocomplexes bound to protein A/G magic beads were washed four times with ice-cold PBS, once with TBST and then eluted with SDS sample buffer. The precipitated proteins were subjected to WB analysis. Co-IP experiments using kidneys from 8-week-old C57BL/6 mice were performed similarly as reported^58^. Briefly, kidney tissue samples were washed with ice-cold PBS and then snap-frozen in liquid nitrogen. Frozen tissue was transferred to a pre-cooled homogenizer for grinding, and an appropriate amount of lysis buffer containing protease inhibitors was added, followed by thorough homogenization on ice. The samples were subsequently lysed at 4 °C for 2 h and then followed by the same procedures as those for mammalian cells.

### Two-electrode voltage clamp electrophysiology

Two-electrode voltage clamp electrophysiological experiments in *Xenopus* oocytes, as reported previously^10,36,59^. Briefly, an electrode made from a capillary glass pipette (Warner Instruments, Hamden, CT) was filled with 3 M KCl, ensuring a tip resistance of 0.3–2.0 MΩ. To reduce endogenous expression of connexin-38 hemichannels, we employed a standard antisense approach. Specifically, 3 ng of antisense thiophosphate oligonucleotide targeting nucleotides 5 to +25 of the cx38 coding region (5′-GCTTTAGTAATTCCCATCCTGCCATGTTTC-3′) (Integrated DNA Technologies) was co-injected with PKD2 RNA. Oocytes injected with antisense oligonucleotide alone served as the control group. The standard Na-containing solution bathing an oocyte is composed of (in mM): 100 NaCl, 2 KCl, 10 Hepes, and pH 7.5. For ion selectivity studies, Na⁺ was replaced with equimolar concentrations of NMDG, Li⁺ or K⁺ in the Na⁺-containing solution, with 2 mM KCl removed. Whole-cell currents and membrane potentials were acquired using a Geneclamp 500B amplifier, a Digidata 1322 A AD/DA converter, and pClamp 10 (Molecular Devices, Union City, CA). Both signals were digitized at 200 μs per sample and filtered through a 2 kHz Bessel filter. The voltage jump protocol used in this study consisted of a holding potential at −50 mV and 200-ms voltage steps in the range of −100 to +100 mV in increments of 20 mV. Data were analyzed using pClamp 10 and plotted using GraphPad Prism 8.3.0 (GraphPad Software, San Diego, CA).

### Surface protein biotinylation in *Xenopus* oocytes and HEK cells

Oocytes were washed three times with ice-cold PBS and then incubated in PBS buffer containing 0.5 mg/mL sulfo-NHS-SS-biotin (Thermo Fisher, 21331) for 1 h at room temperature. Non-reacted biotin was quenched with 1 M NH_4_Cl. Oocytes were then washed with ice cold PBS and harvested in ice-cold CelLytic M lysis buffer (Thermo Fisher, FNN0021) supplemented with proteinase inhibitor cocktail (Thermo Fisher, 78429). After the addition of 30 μL streptavidin (Thermo Fisher, TK275208), the mixture was incubated with gentle agitation overnight at 4 °C, followed by 3–5 washes with ice-cold PBS using centrifugation at 2500 rpm/min (4 °C). The streptavidin-bound surface protein was resuspended in SDS loading buffer and subjected to SDS-PAGE.

Biotin analysis in HEK cells was performed as described before^56^. The cells were harvested and washed three times with pre-cooled PBS. They were then incubated in a PBS buffer containing 0.5 mg/mL sulfo-NHS-SS-biotin for 1 h at room temperature. The remaining steps were the same as for the oocytes.

### Immunofluorescence

Whole-mount immunofluorescence using *Xenopus* oocytes was performed as before^39^. Briefly, oocytes were washed with PBS, fixed in 4% PFA for 15 min, washed three times in PBS and then permeabilized with 0.1% Triton X-100 for 10 min. Oocytes were then blocked in PBS plus 3% skimmed milk for 30 min and incubated with primary antibodies at 4 °C overnight, followed by incubation with Alexa-488-conjugated donkey anti-rabbit secondary antibody (Cell Signaling Technology, 4412S) for 30 min at room temperature. Oocytes were then mounted in Vectashield (Thermo Fisher, S24737) and examined using a confocal laser scanning microscope (Leica SP8, Wetzlar, Germany).

Transfected mammalian cells on glass coverslips were washed three times with ice-cold PBS, fixed with 4% PFA in PBS for 15 min and permeabilized by incubation with 0.1% Triton X-100 for 15 min at room temperature. After blocking with PBS buffer containing 10% goat serum (w/v) for 2 h, samples were incubated with primary antibodies overnight at 4 °C and then with fluorescence-conjugated secondary antibodies in PBS supplemented with 2% FBS and 1% BSA for 2 h at room temperature. A PBS solution containing 1 μg/mL of DAPI (Thermo Fisher, 62248) was prepared for nuclear staining and left to stand for 10 min. After three final washes with ice-cold PBS, the cell-migration samples were fixed onto slides using an anti-fluorescence quenching mounting medium. The prepared slides were dried in the dark and then imaged using a confocal laser scanning microscope (Leica SP8).

### Bimolecular fluorescence complementation

The Venus N- and C-terminal half (VN and VC) was fused to the N-terminal end of human PKD2 (VN-PKD2) and RASSF4 (VC-RASSF4), respectively, for transient expression in HEK cells. HEK cells were inoculated in a 12-well plate containing glass coverslips, and transfection was carried out using polyethyleneimine reagent (at 1:2 ratio) when cells reached 80–90% confluency. After 36–48 h of transfection, sample fixation and preparation were performed according to standard immunofluorescence experimental procedures. Venus fluorescence signals were detected using a confocal laser scanning microscope (Leica SP8) at an excitation wavelength of 488 nm (emission wavelength of 500–550 nm), and all samples were subjected to image acquisition using the same exposure parameters to ensure comparable fluorescence intensities and distributions.

### GST pull-down

The recombinant GST-tagged protein was expressed in BL21-CodonPlus E. coli and purified using glutathione beads according to the manufacturer’s protocol^60^. The purified protein was incubated with lysates from HEK cells overexpressing the Flag-tagged protein overnight at 4 °C. The protein-bound GST-agarose beads were then washed four times with a buffer containing 50 mM Tris/HCl (pH 7.5), 150 mM NaCl, 1 mM EDTA, and 0.5% Nonidet P-40, supplemented with protease inhibitors. Subsequently, the beads were resuspended in SDS-PAGE loading buffer, boiled at 98 °C for 8–10 min, and eluted proteins were subjected to Western blot analysis.

### Gene knockdown, over-expression, and histological analysis in larval zebrafish

Zebrafish experiments were carried out based on a previously described method^24,36^. Briefly, single guide RNA (sgRNA; 3′-AGAACACCGCTCTAGTGCCGCGG-5′) targeting the five coding exons of zebrafish PKD2 was designed and synthesized. The Cas9 (300 pg) was purchased from (Thermo Fisher, A36499). SgRNA, Cas9, and/or mRNA were injected into wild-type AB strain zebrafish embryos at the 1–2 cell stage^61^. Embryos were then grown at 28.5 °C in water supplemented with 50 μg/mL Instant Ocean sea salts. To confirm the efficiency of KD and OE, we extracted genomic DNA and total protein from zebrafish larvae at 5 dpf. The success of genetic manipulation was first verified by DNA gel electrophoresis, followed by sequencing. Furthermore, Western blot analysis was performed to assess the corresponding changes in protein expression.

For histological analyses, larval zebrafish at 5-dpf were anesthetized with tricaine solution and fixed with 4% paraformaldehyde for 24 h at room temperature. After three washes in ice-cold PBS for 15–20 min, larvae were decalcified in EDTA solution (684 mM EDTA, pH 8.0), then washed twice with diethylpyrocarbonate solution (15–20 min each) and consecutively dehydrated in 50, 70, and 95% ethanol each for 3 h. Before paraffin treatment, larval fish were embedded in a Tris Acetate-EDTA buffer containing 1% agarose. Sections were then cut with a Leica Ultracut R ultrathin sectioning machine (Leica EM UC7, Wetzlar, Germany) at a thickness of 1 μm and stained with toluidine blue using a Varistain Gemini ES automatic section stainer (Thermo Fisher, Waltham, MA).

### Behavioral experiments in zebrafish

The experiments were based on the methods described previously^62^. Briefly, 5-dpf larval zebrafish were placed in the wells of a 48-well plate, with each well containing 0.5 mL water (to reduce the vertical swimming space). On the following day, the 48-well plate was placed in a behavioral instrumentation chamber, at 28 °C with the bottom light of the plate on for 90 min as adaptation before measurements. Fish movement trajectories were recorded under the light for 20 min, followed by 5 cycles of dark/light switch, each lasting 10 min, to examine their responses and adaptations to dark and light conditions. Etho Vision XT software (Noldus) was used for recording and analysis.

### Rhodamine-dextran clearance in zebrafish

For in vivo tracing, rhodamine B-conjugated dextran (RD, molecular weight: 10 kDa) was dissolved in ultrapure water to prepare a 50 mg/mL stock solution, which was diluted with autoclaved ultrapure water to a working concentration of 5 mg/mL immediately before injection. Larval zebrafish at 3 dpf were anesthetized with tricaine and immobilized in a pre-grooved agarose mold. Using a microinjection system, approximately 1 nL of the working solution was delivered into the pericardial cavity, and the needle was quickly withdrawn. After injection, fish were first transferred into a small volume of medium for brief observation to confirm resumption of heartbeat and the absence of dye leakage. Fish were then individually placed into wells of a 24-well plate containing 1 mL of fresh medium supplemented with 0.003% N-phenylthiourea (PTU) and incubated at 28.5 °C in the dark for subsequent imaging and analysis^63^.

### Extraction of zebrafish proteins

Ten 5-dpf zebrafish larvae were put in an ice-cold 1.5 mL EP tube with the lysis solution containing protease inhibitor at a ratio of 1:100. The sample was homogenized using a small blender until the sample turned gray but without foam and left on ice for 30 min for protein lysis. After centrifugation at 4 °C and 13,200 rpm for 15 min, the supernatant was aspirated into a new tube, added with the sampling buffer, boiled at 98 °C for 5–10 min, and then cooled in an ice bath before WB analysis.

### Genotype identification in zebrafish

To validate the success of the *pkd2* gene knock out in 3 dpf zebrafish, we extracted genomes from both wild-type and mutant zebrafish. The key steps for genotype identification were as follows: Whole larvae were methanol-fixed, then digested with 10% proteinase K solution at 55 °C for 2 h and 45 min to fully lyse tissues and release genomic DNA. Subsequently, samples were heated to 85 °C for 15 min to inactivate proteinase K, preventing its interference with subsequent PCR reactions. The inactivated digestion products served directly as templates for PCR amplification of the target region. After preliminary confirmation of amplification bands via 2% agarose gel electrophoresis, PCR products were sequenced using specific primers designed for flanking sequences of the target site. Finally, sequence maps were aligned using APE software: if mutant samples exhibited consecutive overlapping peaks or heterozygous double peaks following the expected target site, while wild-type samples showed a clear single peak, the heterozygous knock out was deemed successful.

### Ethical approval

The study was approved by the Ethical Committee for Animal Experiments of the University of Alberta and performed in accordance with the Guidelines for Research with Experimental Animals of the University of Alberta and the Guide for the Care and Use of Laboratory Animals published by the US National Institutes of Health (NIH publication no. 85-23, revised in 1996).

Zebrafish experiments were conducted in accordance with a laboratory animal protocol approved by the Animal Care and Use Committee of Hubei University of Technology. Zebrafish were maintained according to standard protocols (http://ZFIN.org).

### Statistics and reproducibility

WB bands were quantified using ImageJ software (ImageJ 1.51j8). Statistical analyses were performed using GraphPad Prism 9 (GraphPad Software, USA). For comparisons between two groups, two-tailed unpaired or paired Student’s *t* tests were used as appropriate. For comparisons involving more than two groups, one-way analysis of variance (ANOVA) was applied, followed by appropriate post hoc tests. Differences in phenotype distribution among groups were analyzed using chi-squared tests as appropriate. Statistical significance is shown as follows: *p < 0.05, **p < 0.01, ***p < 0.001, and ****p < 0.0001; “ns”, statistically not significant. Depending on the experimental paradigm, data points represent individual cells, electrophysiological recordings, or individual animals, as specified in the corresponding figure legends. For imaging experiments, fluorescence signals were averaged within each biological replicate prior to statistical analysis.

### Reporting summary

Further information on research design is available in the Nature Portfolio Reporting Summary linked to this article.

## Supplementary information

Supplementary Information

Description of Additional Supplementary Files

Supplementary Data

Reporting Summary

## Data Availability

All data supporting the findings of this study are available within the paper and its Supplementary Information files. Source data underlying all figures are provided as Supplementary Data. Uncropped Western blot and gel images are included in the Supplementary information files. Mass spectrometry-based proteomics data have been deposited in the PRIDE database under accession code PXD074819. RNA-sequencing data have been deposited in the NCBI BioProject database under accession code PRJNA1428807.

## References

[1] Bullich, G. et al. A kidney-disease gene panel allows a comprehensive genetic diagnosis of cystic and glomerular inherited kidney diseases. *Kidney Int.* **94**, 363–371 (2018). 29801666 10.1016/j.kint.2018.02.027

[2] Cornec-Le Gall, E., Alam, A. & Perrone, R. D. Autosomal dominant polycystic kidney disease. *Lancet* **393**, 919–935 (2019). 30819518 10.1016/S0140-6736(18)32782-X

[3] Porath, B. et al. Mutations in GANAB, encoding the glucosidase IIalpha subunit, cause autosomal-dominant polycystic kidney and liver disease. *Am. J. Hum. Genet.* **98**, 1193–1207 (2016). 27259053 10.1016/j.ajhg.2016.05.004 PMC4908191

[4] Bergmann, C. et al. Polycystic kidney disease. *Nat. Rev. Dis. Primers* **4**, 50 (2018). 30523303 10.1038/s41572-018-0047-y PMC6592047

[5] Yamaguchi, T. et al. Cyclic AMP activates B-Raf and ERK in cyst epithelial cells from autosomal-dominant polycystic kidneys. *Kidney Int.* **63**, 1983–1994 (2003). 12753285 10.1046/j.1523-1755.2003.00023.x

[6] Yamaguchi, T., Reif, G. A., Calvet, J. P. & Wallace, D. P. Sorafenib inhibits cAMP-dependent ERK activation, cell proliferation, and in vitro cyst growth of human ADPKD cyst epithelial cells. *Am. J. Physiol. Ren. Physiol.* **299**, F944–F951 (2010). 10.1152/ajprenal.00387.2010 PMC2980397 20810616

[7] Tsilosani, A. et al. Pkd2 deficiency in embryonic aqp2+ progenitor cells is sufficient to cause severe polycystic kidney disease. *J. Am. Soc. Nephrol.* **35**, 398–409 (2024). 38254271 10.1681/ASN.0000000000000309 PMC11000715

[8] Hopp, K. et al. Functional polycystin-1 dosage governs autosomal dominant polycystic kidney disease severity. *J. Clin. Investig.* **122**, 4257–4273 (2012). 23064367 10.1172/JCI64313 PMC3484456

[9] Grieben, M. et al. Structure of the polycystic kidney disease TRP channel polycystin-2 (PC2). *Nat. Struct. Mol. Biol.* **24**, 114–122 (2017). 27991905 10.1038/nsmb.3343

[10] Zheng, W. et al. Hydrophobic pore gates regulate ion permeation in polycystic kidney disease 2 and 2L1 channels. *Nat. Commun.* **9**, 2302 (2018). 29899465 10.1038/s41467-018-04586-x PMC5998024

[11] Douguet, D., Patel, A. & Honore, E. Structure and function of polycystins: insights into polycystic kidney disease. *Nat. Rev. Nephrol.* **15**, 412–422 (2019). 30948841 10.1038/s41581-019-0143-6

[12] Shen, P. S. et al. The structure of the polycystic kidney disease channel PKD2 in lipid nanodiscs. *Cell* **167**, 763–773 (2016). 27768895 10.1016/j.cell.2016.09.048 PMC6055481

[13] Wang, S., Kang, Y. & Xie, H. PKD2: an important membrane protein in organ development. *Cells* **13**, 1722 (2024). 10.3390/cells13201722 PMC11506562 39451240

[14] Formica, C. & Peters, D. J. M. Molecular pathways involved in injury-repair and ADPKD progression. *Cell Signal.* **72**, 109648 (2020). 32320858 10.1016/j.cellsig.2020.109648

[15] Su, Q. et al. Structure of the human PKD1-PKD2 complex. *Science* **361**, eaat9819 (2018). 10.1126/science.aat9819 30093605

[16] Yu, Y. et al. Structural and molecular basis of the assembly of the TRPP2/PKD1 complex. *Proc. Natl. Acad. Sci. USA* **106**, 11558–11563 (2009). 19556541 10.1073/pnas.0903684106 PMC2710685

[17] Kobori, T., Smith, G. D., Sandford, R. & Edwardson, J. M. The transient receptor potential channels TRPP2 and TRPC1 form a heterotetramer with a 2:2 stoichiometry and an alternating subunit arrangement. *J. Biol. Chem.* **284**, 35507–35513 (2009). 19850920 10.1074/jbc.M109.060228 PMC2790980

[18] Kottgen, M. et al. TRPP2 and TRPV4 form a polymodal sensory channel complex. *J. Cell Biol.* **182**, 437–447 (2008). 18695040 10.1083/jcb.200805124 PMC2500130

[19] Kwak, M. et al. The mutual interaction of TRPC5 channel with polycystin proteins. *Korean J. Physiol. Pharmacol.* **29**, 93–108 (2025). 39523478 10.4196/kjpp.24.265 PMC11694000

[20] Lehtonen, S., Zhao, F. & Lehtonen, E. CD2-associated protein directly interacts with the actin cytoskeleton. *Am. J. Physiol. Ren. Physiol.* **283**, F734–F743 (2002). 10.1152/ajprenal.00312.2001 12217865

[21] Li, Q. et al. Alpha-actinin associates with polycystin-2 and regulates its channel activity. *Hum. Mol. Genet.* **14**, 1587–1603 (2005). 15843396 10.1093/hmg/ddi167

[22] Li, Y., Wright, J. M., Qian, F., Germino, G. G. & Guggino, W. B. Polycystin 2 interacts with type I inositol 1,4,5-trisphosphate receptor to modulate intracellular Ca^2+^ signaling. *J. Biol. Chem.* **280**, 41298–41306 (2005). 16223735 10.1074/jbc.M510082200

[23] Liang, G. et al. Polycystin-2 down-regulates cell proliferation via promoting PERK-dependent phosphorylation of eIF2alpha. *Hum. Mol. Genet.* **17**, 3254–3262 (2008). 18664456 10.1093/hmg/ddn221

[24] Liu, X. et al. Regulation of PKD2 channel function by TACAN. *J. Physiol.* **601**, 83–98 (2023). 36420836 10.1113/JP283895

[25] Chow, L. S., Lo, K., Kwong, J., Wong, A. Y. & Huang, D. P. Aberrant methylation of RASSF4/AD037 in nasopharyngeal carcinoma. *Oncol. Rep.* **12**, 781–787 (2004). 15375500

[26] Fernandes, M. S., Carneiro, F., Oliveira, C. & Seruca, R. Colorectal cancer and RASSF family—a special emphasis on RASSF1A. *Int. J. Cancer* **132**, 251–258 (2013). 22733432 10.1002/ijc.27696

[27] Iwasa, H. et al. Characterization of RSF-1, the *Caenorhabditis elegans* homolog of the Ras-association domain family protein 1. *Exp. Cell Res.* **319**, 1–11 (2013). 23103556 10.1016/j.yexcr.2012.10.008

[28] Volodko, N., Gordon, M., Salla, M., Ghazaleh, H. A. & Baksh, S. RASSF tumor suppressor gene family: biological functions and regulation. *FEBS Lett.* **588**, 2671–2684 (2014). 24607545 10.1016/j.febslet.2014.02.041

[29] De Smedt, E. et al. Loss of RASSF4 expression in multiple myeloma promotes RAS-driven malignant progression. *Cancer Res.* **78**, 1155–1168 (2018). 29259009 10.1158/0008-5472.CAN-17-1544

[30] Vos, M. D., Martinez, A., Ellis, C. A., Vallecorsa, T. & Clark, G. J. The pro-apoptotic Ras effector nore1 may serve as a Ras-regulated tumor suppressor in the lung. *J. Biol. Chem.* **278**, 21938–21943 (2003). 12676952 10.1074/jbc.M211019200

[31] Liu, A. et al. A “Janus” face of the RASSF4 signal in cell fate. *J. Cell. Physiol.* **237**, 466–479 (2022). 34553373 10.1002/jcp.30592

[32] Eckfeld, K. et al. RASSF4/AD037 is a potential Ras effector/tumor suppressor of the RASSF family. *Cancer Res.* **64**, 8688–8693 (2004). 15574778 10.1158/0008-5472.CAN-04-2065

[33] Xu, C. et al. RASSF4 attenuates metabolic dysfunction-associated steatotic liver disease progression via hippo signaling and suppresses hepatocarcinogenesis. *Cell. Mol. Gastroenterol. Hepatol.* **18**, 101348 (2024). 38697356 10.1016/j.jcmgh.2024.04.005 PMC11217689

[34] Roux, K. J., Kim, D. I., Raida, M. & Burke, B. A promiscuous biotin ligase fusion protein identifies proximal and interacting proteins in mammalian cells. *J. Cell Biol.* **196**, 801–810 (2012). 22412018 10.1083/jcb.201112098 PMC3308701

[35] Cho, K. F. et al. Split-TurboID enables contact-dependent proximity labeling in cells. *Proc. Natl. Acad. Sci. USA* **117**, 12143–12154 (2020). 32424107 10.1073/pnas.1919528117 PMC7275672

[36] Arif Pavel, M. et al. Function and regulation of TRPP2 ion channel revealed by a gain-of-function mutant. *Proc. Natl. Acad. Sci. USA* **113**, E2363–E2372 (2016). 27071085 10.1073/pnas.1517066113 PMC4855601

[37] Sun, Z. et al. A genetic screen in zebrafish identifies cilia genes as a principal cause of cystic kidney. *Development* **131**, 4085–4093 (2004). 15269167 10.1242/dev.01240

[38] Zheng, W. et al. Far upstream element-binding protein 1 binds the 3’ untranslated region of PKD2 and suppresses its translation. *J. Am. Soc. Nephrol.* **27**, 2645–2657 (2016). 26839368 10.1681/ASN.2015070836 PMC5004656

[39] Zheng, W. et al. Direct binding between pre-s1 and TRP-like domains in TRPP channels mediates gating and functional regulation by PIP2. *Cell Rep.* **22**, 1560–1573 (2018). 29425510 10.1016/j.celrep.2018.01.042 PMC6483072

[40] Su, L. et al. Ganoderma triterpenes retard renal cyst development by downregulating Ras/MAPK signaling and promoting cell differentiation. *Kidney Int.* **92**, 1404–1418 (2017). 28709639 10.1016/j.kint.2017.04.013

[41] Dagorn, P. G. et al. A novel direct adenosine monophosphate kinase activator ameliorates disease progression in preclinical models of autosomal dominant polycystic kidney disease. *Kidney Int.* **103**, 917–929 (2023). 36804411 10.1016/j.kint.2023.01.026

[42] Tan, M. et al. Novel inhibitors of nuclear transport cause cell cycle arrest and decrease cyst growth in ADPKD associated with decreased CDK4 levels. *Am. J. Physiol. Ren. Physiol.* **307**, F1179–F1186 (2014). 10.1152/ajprenal.00406.2014 PMC4254973 25234309

[43] Chen, Y., Chang, C., Lee, W. & Liou, J. RASSF4 controls SOCE and ER-PM junctions through regulation of PI(4,5)P(2). *J. Cell Biol.* **216**, 2011–2025 (2017). 28600435 10.1083/jcb.201606047 PMC5496610

[44] Makbul, C. et al. Structural and thermodynamic characterization of nore1-SARAH: a small, helical module important in signal transduction networks. *Biochemistry* **52**, 1045–1054 (2013). 23331050 10.1021/bi3014642

[45] Cai, R. et al. Autoinhibition of TRPV6 channel and regulation by PIP2. *iScience* **23**, 101444 (2020). 32829285 10.1016/j.isci.2020.101444 PMC7452202

[46] Chang, M. et al. Inhibition of the P2X7 receptor reduces cystogenesis in PKD. *J. Am. Soc. Nephrol.* **22**, 1696–1706 (2011). 21636640 10.1681/ASN.2010070728 PMC3171940

[47] Sussman, C. R. et al. Phosphodiesterase 1A modulates cystogenesis in zebrafish. *J. Am. Soc. Nephrol.* **25**, 2222–2230 (2014). 24700876 10.1681/ASN.2013040421 PMC4178429

[48] Meng, J. et al. Ganoderic acid A is the effective ingredient of ganoderma triterpenes in retarding renal cyst development in polycystic kidney disease. *Acta Pharmacol. Sin.* **41**, 782–790 (2020). 31911637 10.1038/s41401-019-0329-2 PMC7468358

[49] Zhou, H. et al. Ginkgolide B inhibits renal cyst development in in vitro and in vivo cyst models. *Am. J. Physiol. Ren. Physiol.* **302**, F1234–F1242 (2012). 10.1152/ajprenal.00356.2011 PMC3362062 22338085

[50] Chapin, H. C. & Caplan, M. J. The cell biology of polycystic kidney disease. *J. Cell Biol.* **191**, 701–710 (2010). 21079243 10.1083/jcb.201006173 PMC2983067

[51] Mariotti, V., Fiorotto, R., Cadamuro, M., Fabris, L. & Strazzabosco, M. New insights on the role of vascular endothelial growth factor in biliary pathophysiology. *JHEP Rep.* **3**, 100251 (2021). 34151244 10.1016/j.jhepr.2021.100251 PMC8189933

[52] Garcia-Navarrete, C. et al. PKD2 regulates autophagy and forms a protein complex with BECN1 at the primary cilium of hypothalamic neuronal cells. *Biochim. Biophys. Acta Mol. Basis Dis.* **1870**, 167256 (2024). 38782303 10.1016/j.bbadis.2024.167256

[53] Tian, R. et al. The dual role of RASSF4 in tumorigenesis: mechanisms and epigenetic targeting strategies. *Biology* **14**, 1289 (2025). 10.3390/biology14091289 PMC12467136 41007433

[54] Grantham, J. J. Polycystic kidney disease: neoplasia in disguise. *Am. J. Kidney Dis.* **15**, 110–116 (1990). 2405652 10.1016/s0272-6386(12)80507-5

[55] Jdiaa, S. S. et al. A systematic review of reported outcomes in ADPKD studies. *Kidney Int. Rep.* **7**, 1964–1979 (2022). 36090492 10.1016/j.ekir.2022.06.012 PMC9459055

[56] Huang, Y. et al. The LCK-14-3-3zeta-TRPM8 axis regulates TRPM8 function/assembly and promotes pancreatic cancer malignancy. *Cell Death Dis.* **13**, 524 (2022). 35665750 10.1038/s41419-022-04977-5 PMC9167300

[57] Zhang, R. et al. RUNDC1 inhibits autolysosome formation and survival of zebrafish via clasping ATG14-STX17-SNAP29 complex. *Cell Death Differ.* **30**, 2231–2248 (2023). 37684417 10.1038/s41418-023-01215-z PMC10589263

[58] Li, K. et al. Hrd1-mediated ACLY ubiquitination alleviate NAFLD in db/db mice. *Metab. Clin. Exp.* **114**, 154349 (2021). 32888949 10.1016/j.metabol.2020.154349

[59] Zheng, W. et al. Author correction: hydrophobic pore gates regulate ion permeation in polycystic kidney disease 2 and 2L1 channels. *Nat. Commun.* **10**, 1452 (2019). 30914650 10.1038/s41467-019-09422-4 PMC6435729

[60] Zhou, C. et al. Phosphorylated STYK1 restrains the inhibitory role of EGFR in autophagy initiation and EGFR-TKIs sensitivity. *Cell Insight* **1**, 100045 (2022). 37192859 10.1016/j.cellin.2022.100045 PMC10120315

[61] Xin, Y. & Duan, C. Microinjection of antisense morpholinos, CRISPR/Cas9 RNP, and RNA/DNA into zebrafish embryos. *Methods Mol. Biol.* **1742**, 205–211 (2018). 29330802 10.1007/978-1-4939-7665-2_18

[62] Duan, W. et al. Deficiency of DDX3X results in neurogenesis defects and abnormal behaviors via dysfunction of the notch signaling. *Proc. Natl. Acad. Sci. USA* **121**, e1890794175 (2024). 10.1073/pnas.2404173121 PMC11551356 39471229

[63] Christou-Savina, S., Beales, P. L. & Osborn, D. P. S. Evaluation of zebrafish kidney function using a fluorescent clearance assay. *J. Vis. Exp.* **96**, e52540 (2015). 10.3791/52540 PMC4354652 25742415

[64] Staudner, T. et al. Disease-associated missense mutations in the pore loop of polycystin-2 alter its ion channel function in a heterologous expression system. *J. Biol. Chem.* **300**, 107574 (2024). 39009345 10.1016/j.jbc.2024.107574 PMC11630642

