# Supplementary material for: Regulation of the PKD2 channel function and associated disease phenotypes by RASSF4
Source: Commun Biol. 2026 May 15;9:1035. doi: 10.1038/s42003-026-10242-8 (PMC13434659; doi:10.1038/s42003-026-10242-8)
Supplement: Supplementary file 1 [file 42003_2026_10242_MOESM1_ESM.pdf]

Supplementary Information

Regulation of the PKD2 channel function and associated disease phenotypes by RASSF4

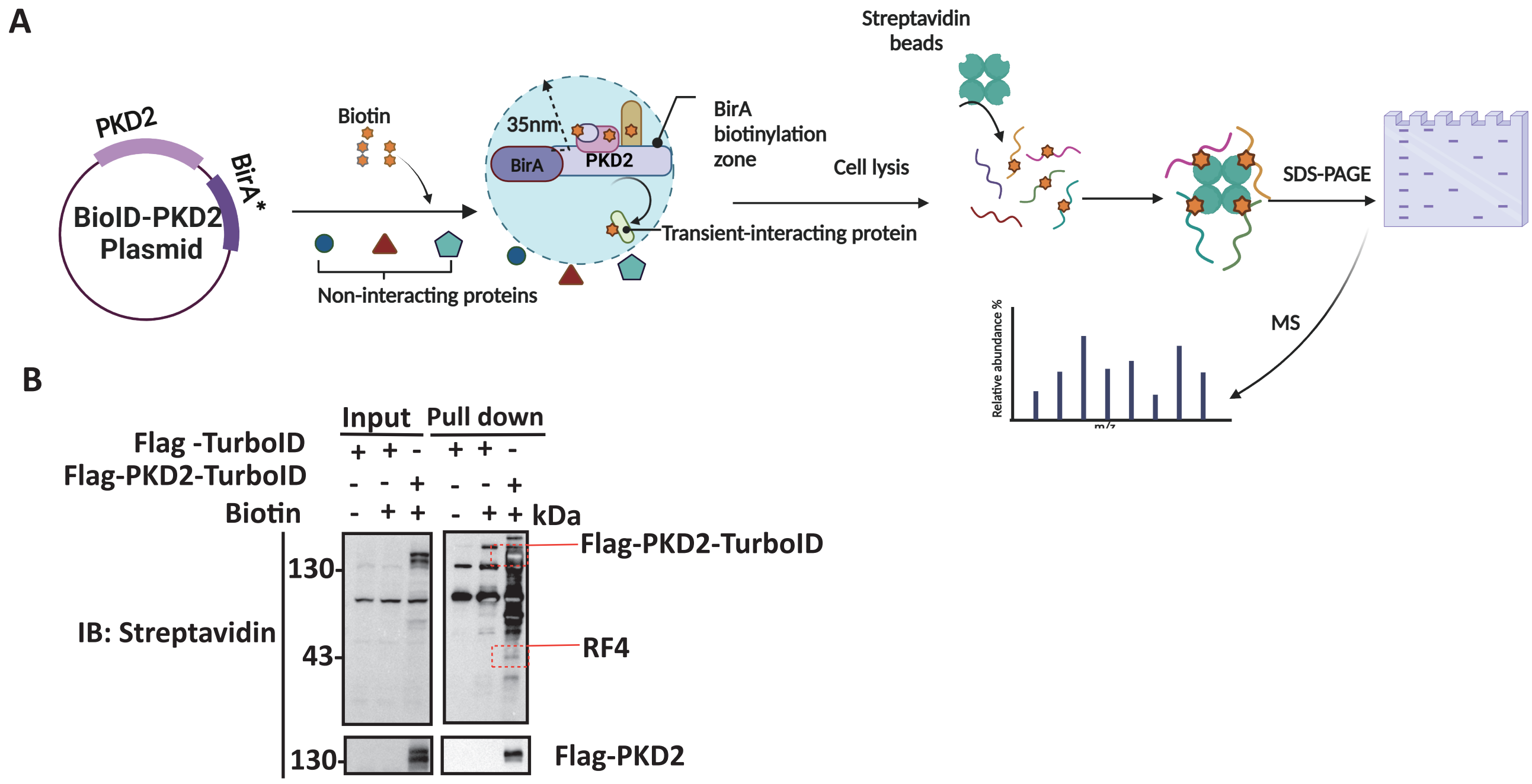

Supplementary Figure 1. Identification of RF4 as a potential binding partner of human PKD2 as a bait from neighboring protein biotin labeling assays and mass spectrometry (MS) analysis. A. Schematic description of the main steps of a biotin neighbor labeling assay plus MS. B. Transient expression of human PKD2 in HEK cells and detection by WB. Biotin was added 6 hr before harvesting for WB and MS analysis.

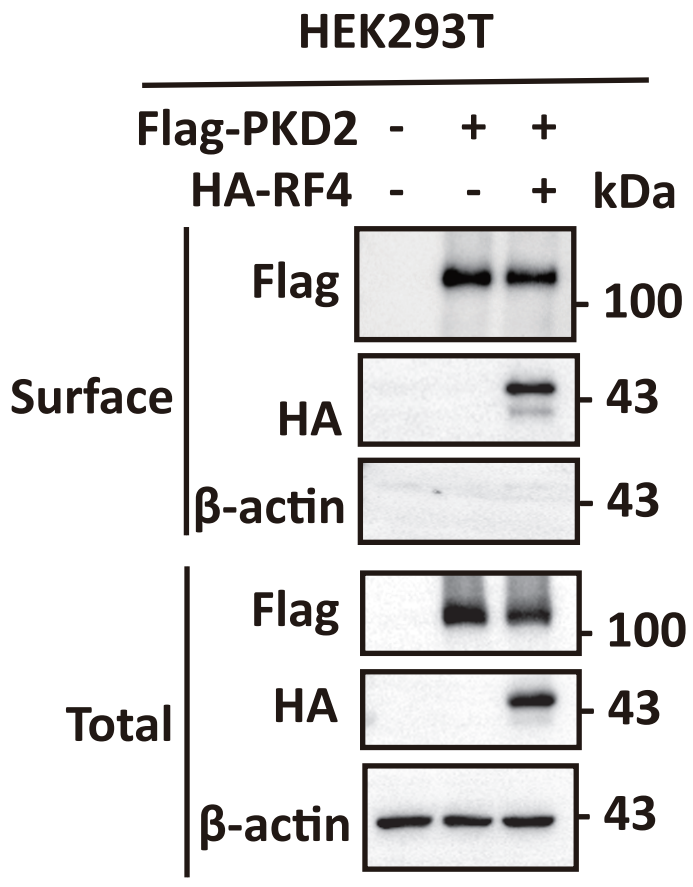

Supplementary Figure 2. Effect of RASSF4 on the surface membrane expression of PKD2. Representative data showing the surface and total expression of PKD2 and RASSF4 in HEK cells revealed by a surface biotinylation assay.

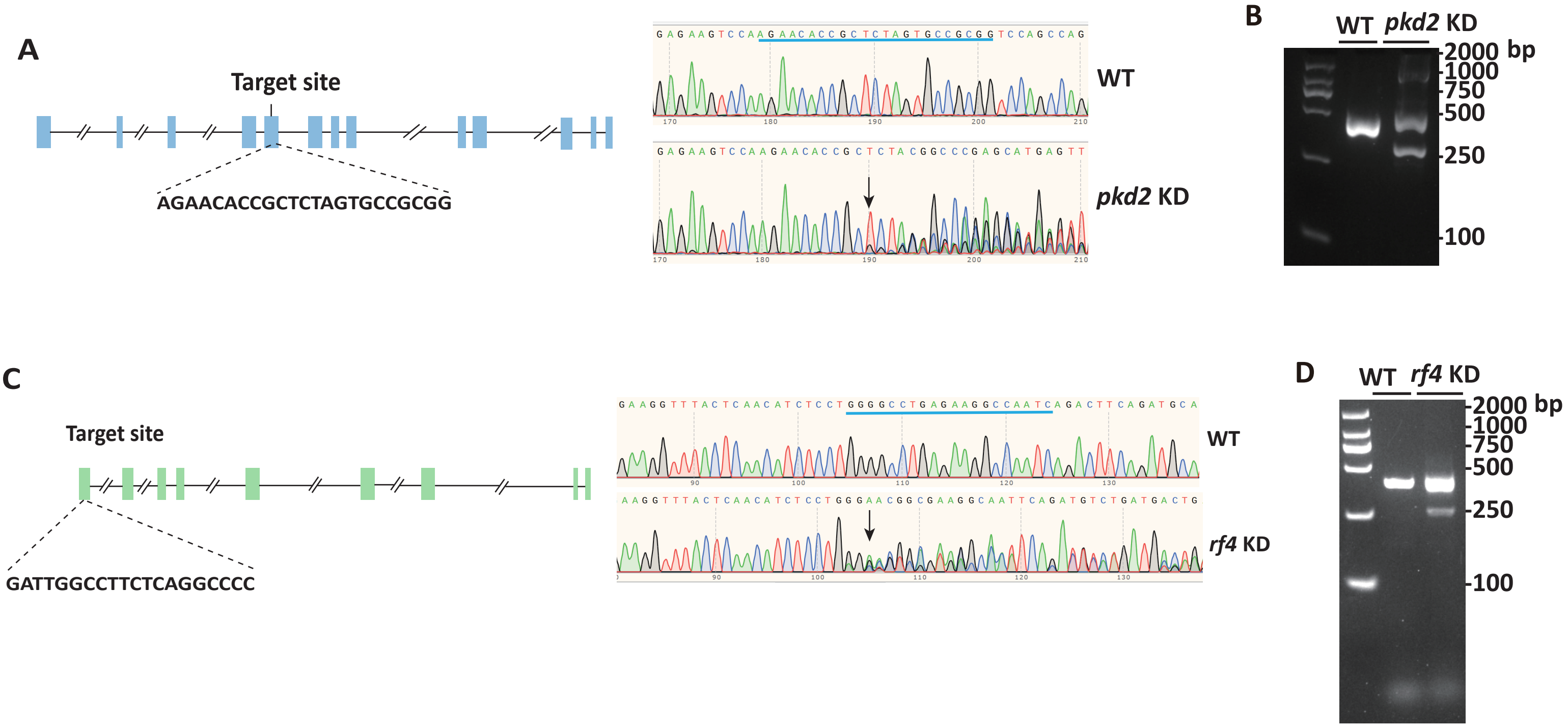

Supplementary Figure 3. Effectiveness of *pkd2* and *rassf4* KD by CRISPR/Cas9 in larval zebrafish. A. Left panel, schematic representation of *pkd2* genomic DNA with indicated exons (blue bar), introns (lines), target site, and sgRNA sequence for KD. Right panel, *pkd2* sequencing in 3-dpf larval zebrafish with WT *pkd2* and *pkd2* KD. The black arrow indicates the cleavage site. B. Genotypic verification of *pkd2* KD by PCR amplification. The double bands indicate the presence of insertion/deletion (Indel) mutations. C. Left panel, schematic diagram of the *rassf4* genomic DNA, annotated with exons (green bar), introns (lines), target site, and sgRNA sequence for KD. Right panel, *rassf4* sequencing in 3-dpf larval zebrafish with WT *rassf4* and *rassf4* KD. The black arrow indicates the cleavage site. D. Genotypic verification of *rassf4* KD by PCR amplification.

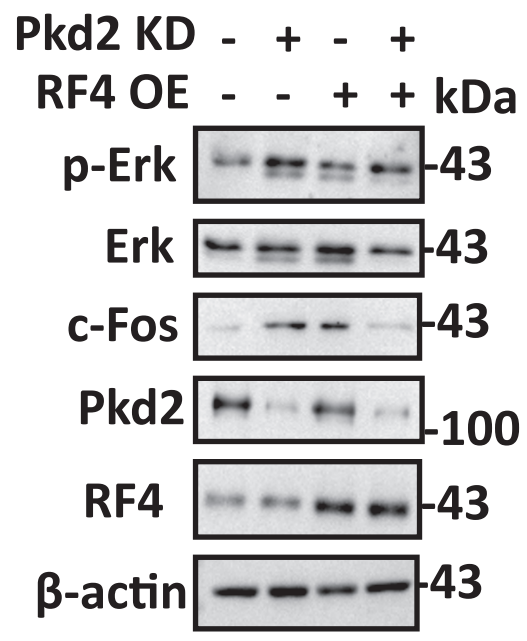

Supplementary Figure 4. Effect of RASSF4 on the RAS/MAPK signaling activity. Representative WB data with Pkd2 KD or RASSF4 OE in 5-dpf zebrafish, showing the expression of important factors associated with the signaling pathway.

Figure 1

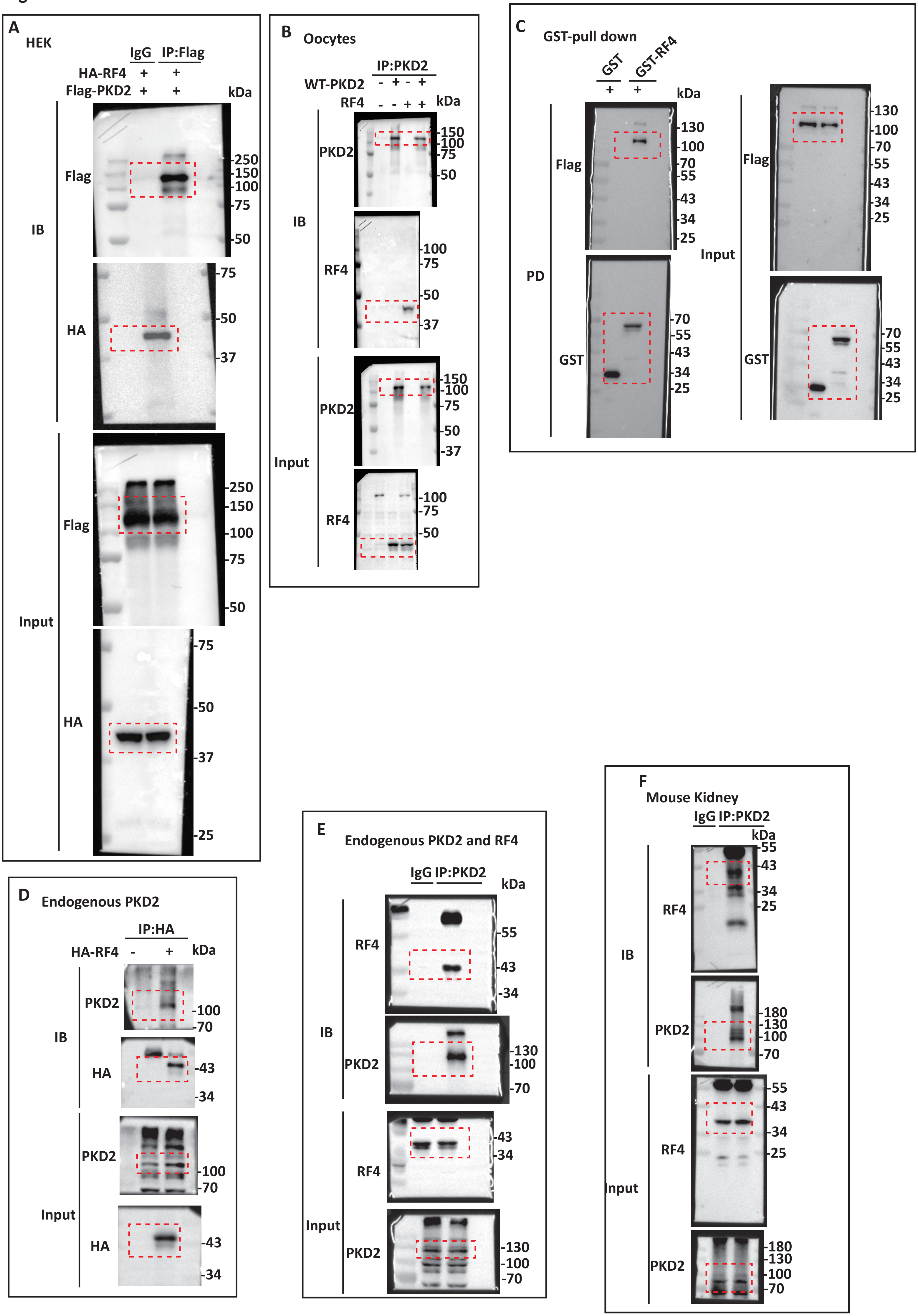
A
HEK
IgG
IP:Flag
HA-RF4
Flag-PKD2
kDa
IB
Flag
HA
Input
B
Oocytes
IP:PKD2
WT-PKD2
RF4
PKD2
C
GST-pull down
GST
GST-RF4
PD
GST
D
Endogenous PKD2
IP:HA
E
Endogenous PKD2 and RF4
IgG IP:PKD2
F
Mouse Kidney
IgG IP:PKD2

Supplementary Figure 5. Original immunoblot scans corresponding to Fig. 1. Dashed boxes indicate the regions shown in the main figure.

Figure 2

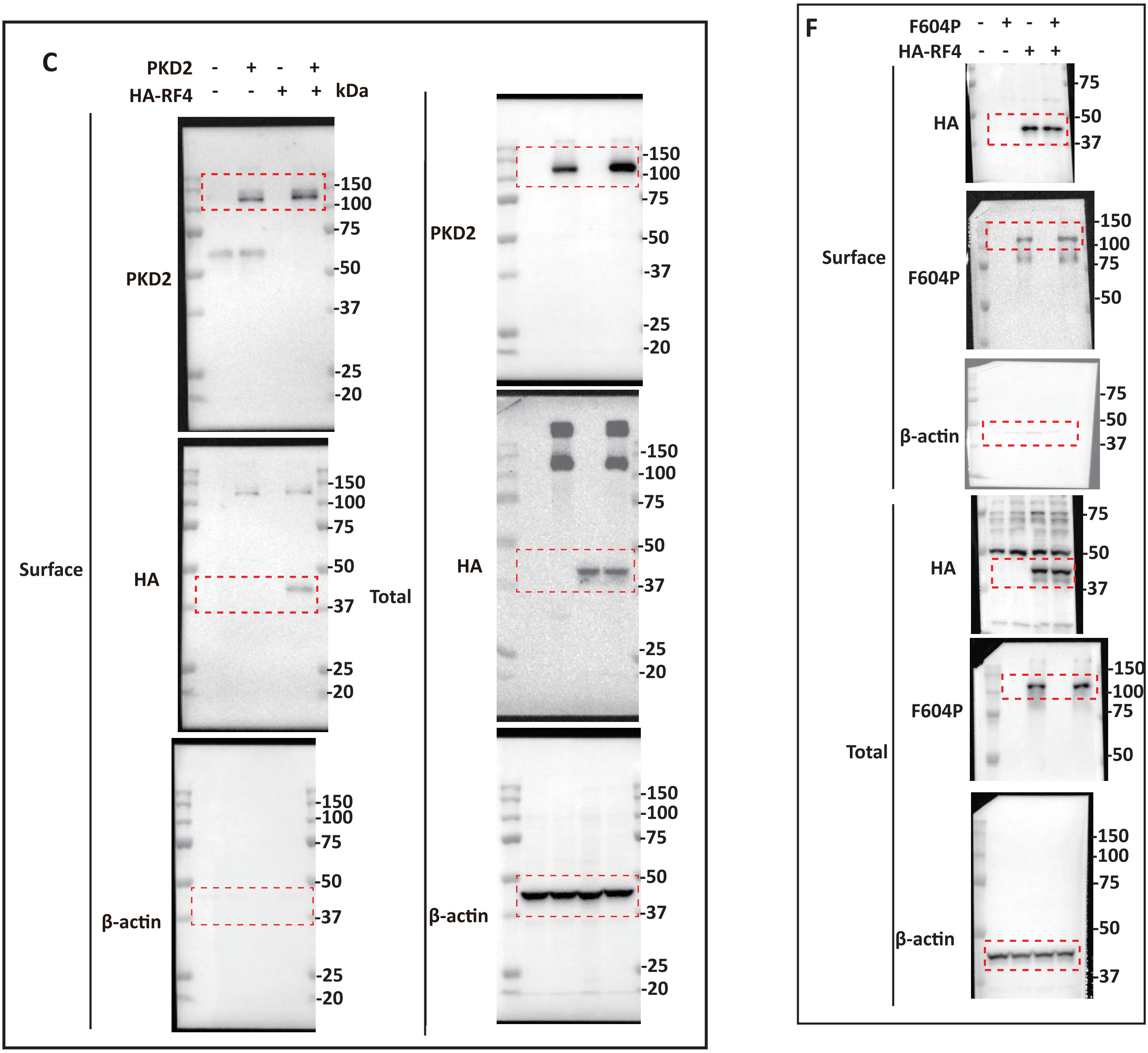

Supplementary Figure 6. Original immunoblot scans corresponding to Fig. 2. Dashed boxes indicate the regions shown in the main figure.

Figure 3

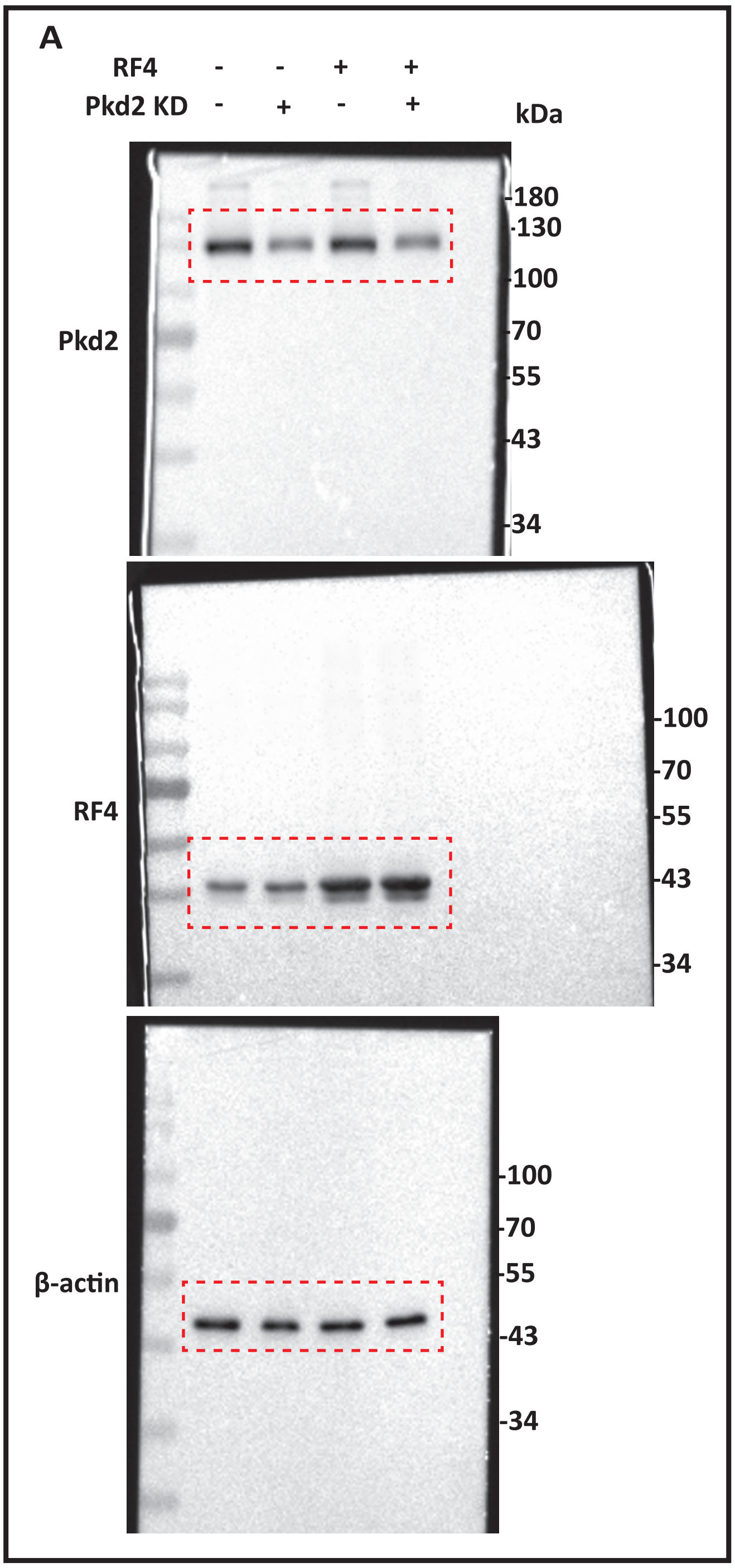

Supplementary Figure 7. Original immunoblot scans corresponding to Fig. 3. Dashed boxes indicate the regions shown in the main figure.

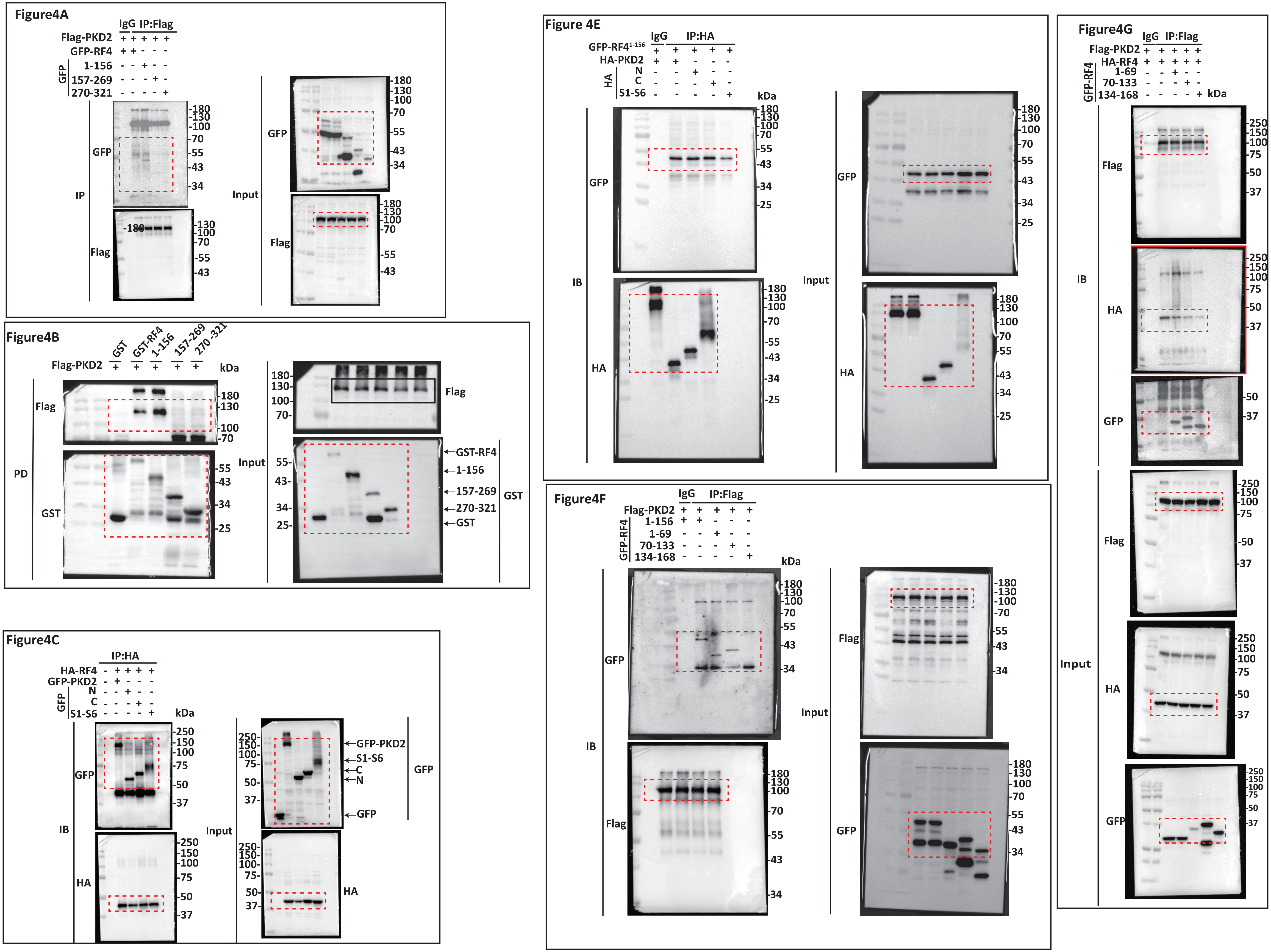

Figure4A
IgG IP:Flag
Flag-PKD2
GFP-RF4
GFP 1-156
157-269
270-321
IP
GFP
Flag
Input
Figure4B
GST
GST-RF4
1-156
157-269
270-321
Flag-PKD2
kDa
PD
Flag
GST
Input
GST-RF4
GST
Figure4C
IP:HA
HA-RF4
GFP-PKD2
GFP N
C
S1-S6
kDa
IB
GFP
HA
Input
GFP-PKD2
S1-S6
C
N
GFP
Figure 4E
IgG IP:HA
GFP-RF4 1-156
HA-PKD2
HA N
C
S1-S6
kDa
IB
GFP
HA
Input
Figure4F
IgG IP:Flag
Flag-PKD2
GFP-RF4 1-156
1-69
70-133
134-168
kDa
IB
GFP
Flag
Input
Figure4G
IgG IP:Flag
Flag-PKD2
HA-RF4
GFP-RF4 1-69
70-133
134-168
kDa
IB
Flag
HA
GFP
Input

Supplementary Figure 8. Original immunoblot scans corresponding to Fig. 4. Dashed boxes indicate the regions shown in the main figure.

Figure 5

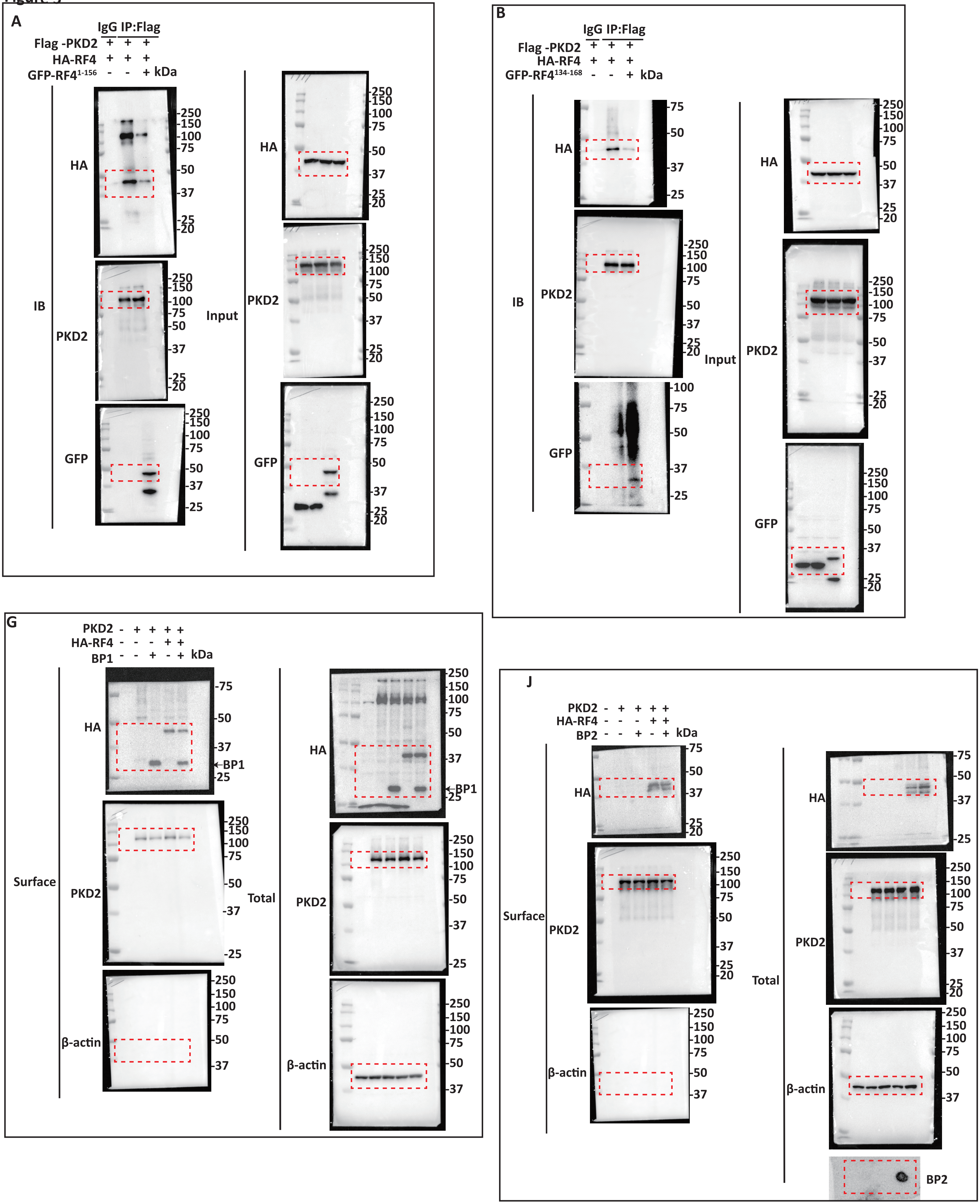
A
IgG IP:Flag
Flag -PKD2 + + +
HA-RF4 + + +
GFP-RF4 1-156 - - + kDa
IB
HA
PKD2
GFP
Input
B
IgG IP:Flag
Flag -PKD2 + + +
HA-RF4 + + +
GFP-RF4 134-168 - - + kDa
IB
HA
PKD2
GFP
Input
G
PKD2 - + + + +
HA-RF4 - - - + +
BP1 - - + - + kDa
Surface
HA
←BP1
PKD2
β-actin
Total
J
PKD2 - + + + +
HA-RF4 - - - + +
BP2 - - + - + kDa
Surface
HA
PKD2
β-actin
Total
BP2

Supplementary Figure 9. Original immunoblot scans corresponding to Fig. 5. Dashed boxes indicate the regions shown in the main figure.

Figure 7

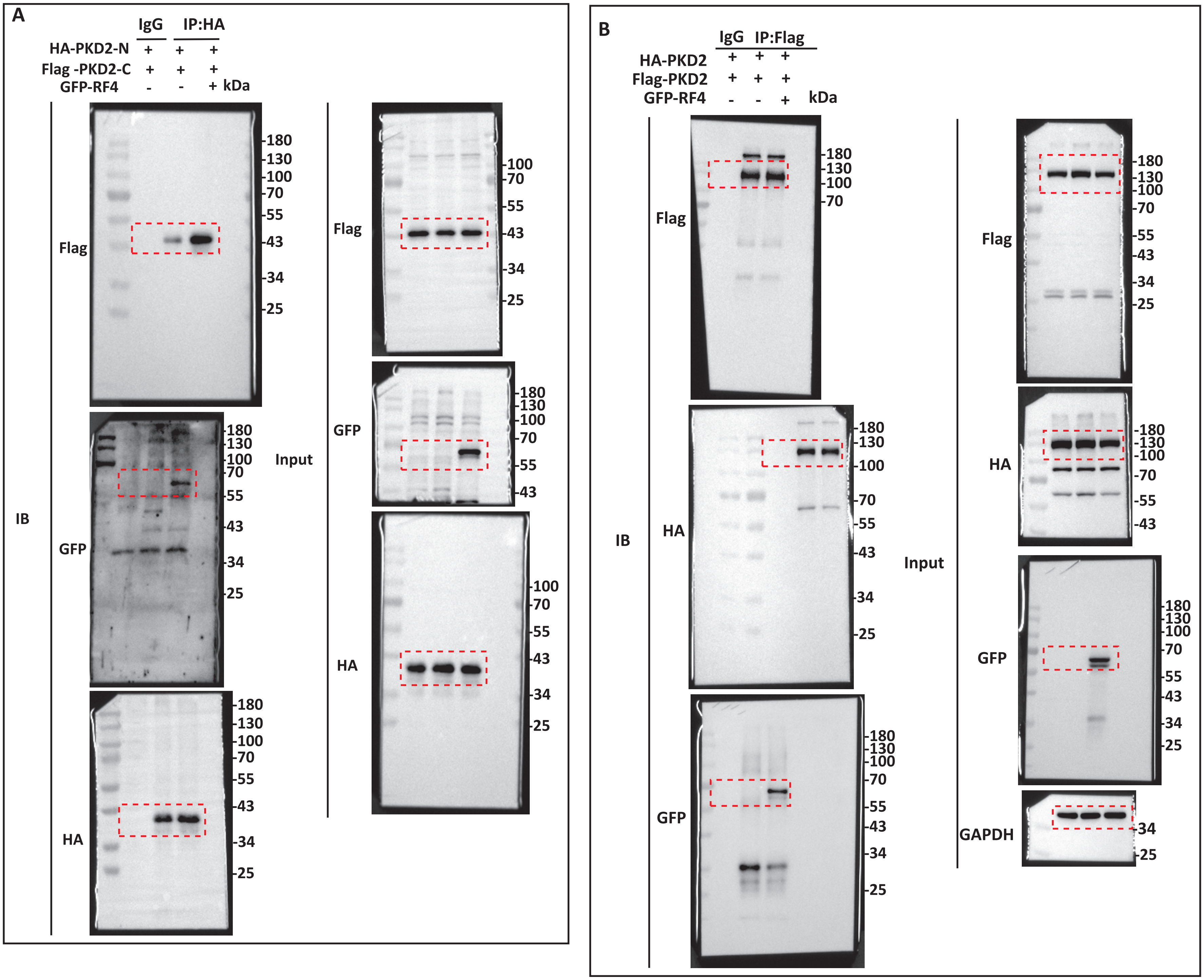

Supplementary Figure 10. Original immunoblot scans corresponding to Fig. 7 A and B. Dashed boxes indicate the regions shown in the main figure.

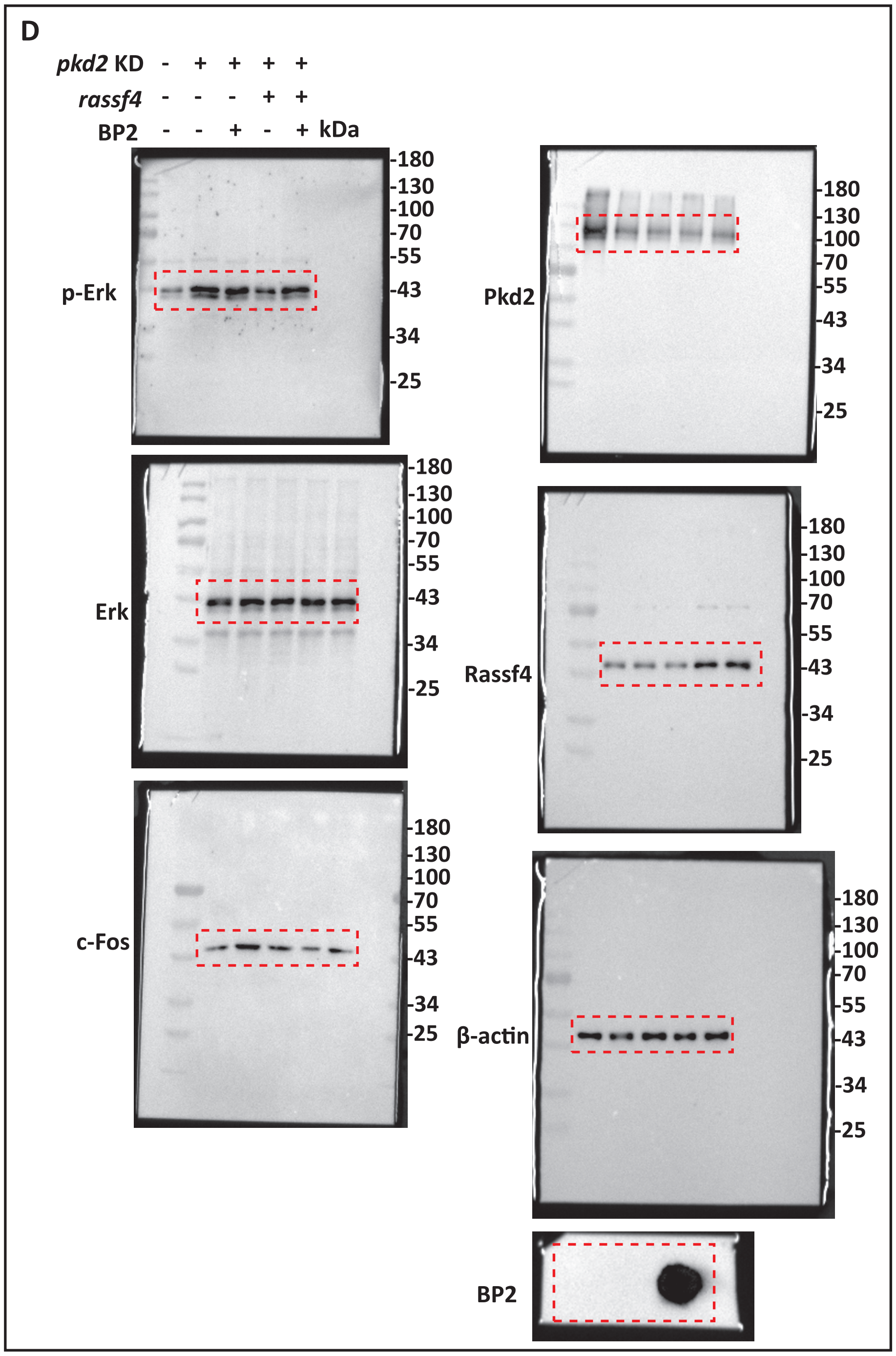

Supplementary Figure 11. Original immunoblot scans corresponding to Fig. 7 D. Dashed boxes indicate the regions shown in the main figure.

Figure S1

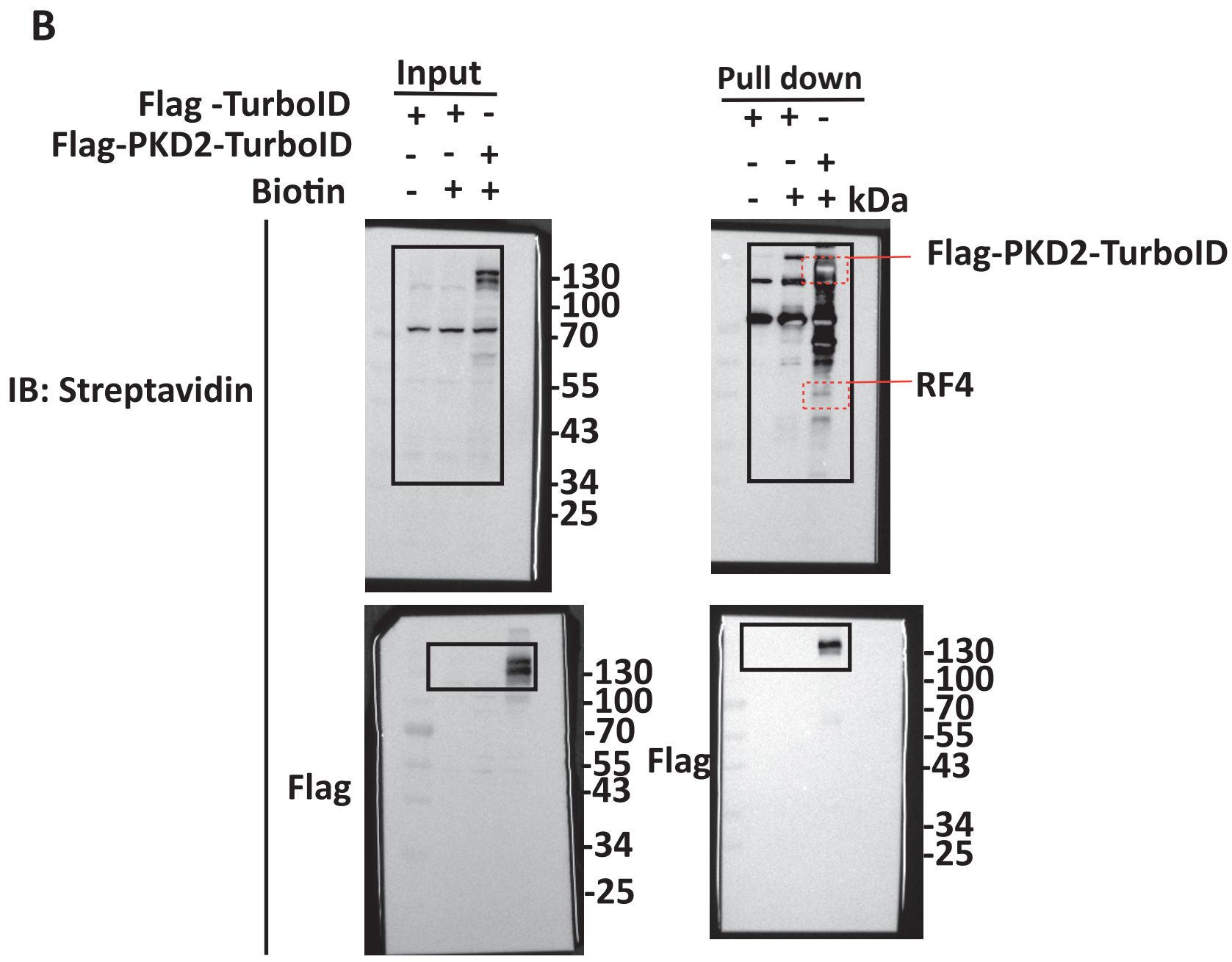

Supplementary Figure 12. Original immunoblot scans corresponding to Fig. S1. Dashed boxes indicate the regions shown in Fig. S1.

Figure S2

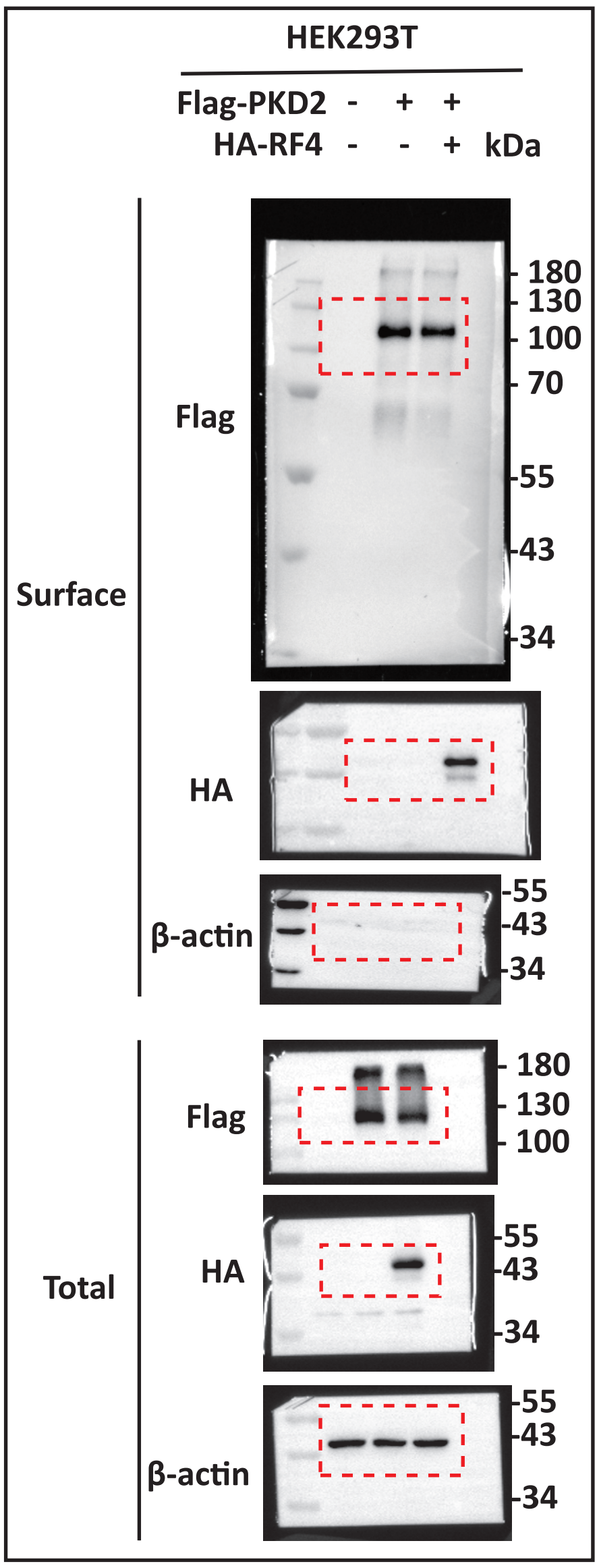

Supplementary Figure 13. Original immunoblot scans corresponding to Fig. S2. Dashed boxes indicate the regions shown in Fig. S2.

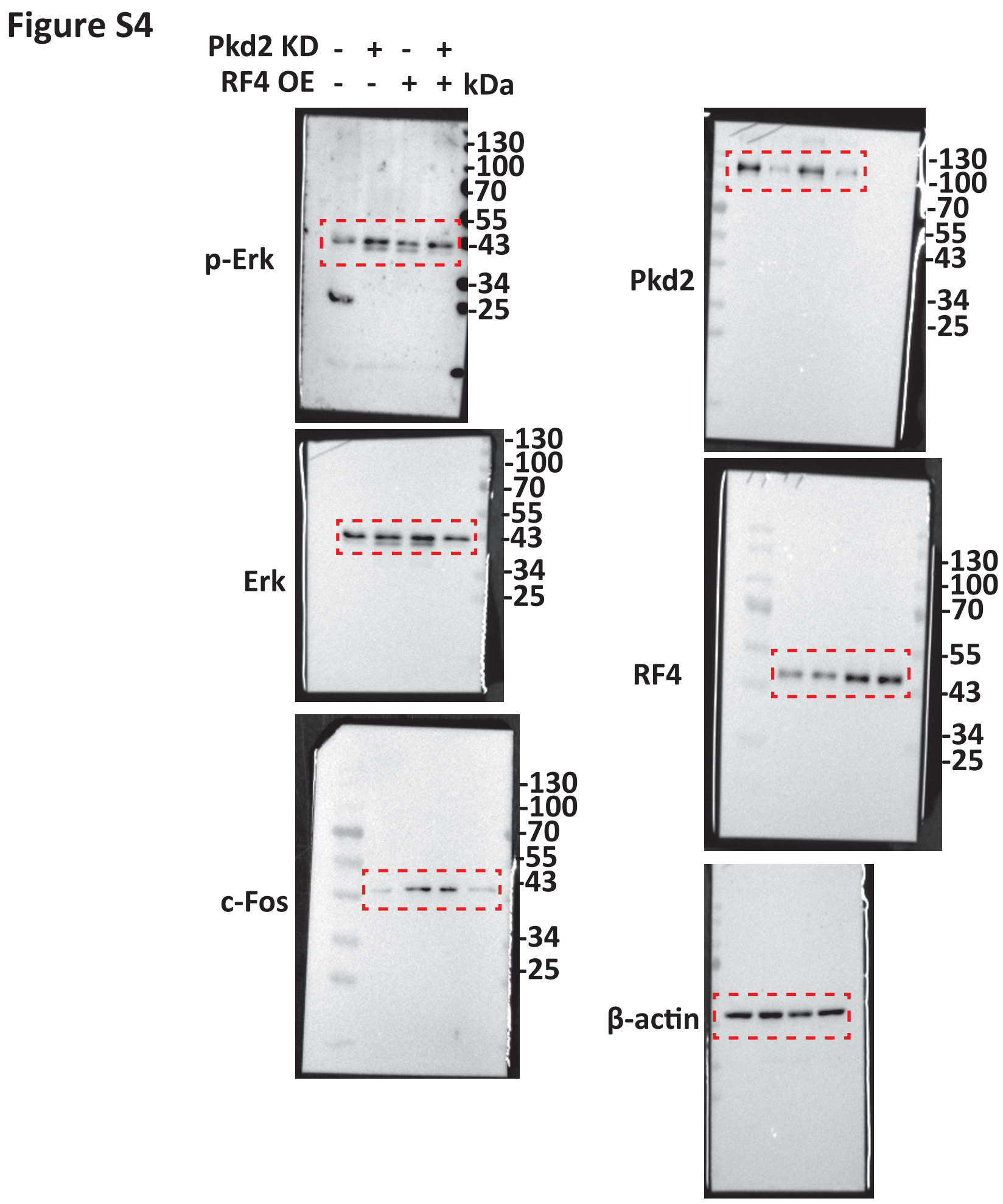

Supplementary Figure 14. Original immunoblot scans corresponding to Fig. S4. Dashed boxes indicate the regions shown in Fig. S4.

Supplementary Table 1. Experimental antibody information

Antibody name	brand	product number	dilution ratio
anti-Rabbit PKD2	Proteintech	19126-1-AP	1:2000
anti-Rabbit RASSF4	Thermo Fisher	PA5-103245	1:2000
anti- Mouse GAPDH	Proteintech	60004-1-Ig	1:5000
anti-Mouse β-actin	Santa Cruz Biotechnology	sc-47778	1:5000
anti-Mouse HA	Sigma	05-904	1:2000
anti-Rabbit HA	Proteintech	51064-2-AP	1:5000
anti-Mouse DYKDDDDK	MBL	M185-3S	1:5000
anti-Rabbit DYKDDDDK	Proteintech	20543-1-AP	1:5000
anti-Rabbit-ERK	Cell signaling technology	4695T	1:1000
anti-Mouse-p-ERK	Cell signaling technology	4377T	1:1000
anti-Rabbit GFP	Proteintech	50430-2-AP	1:2000
anti-Mouse GFP	Proteintech	66002-1-Ig	1:20000
HRP-conjugated Streptavidin	Proteintech	SA00001-0	1:3000
anti-Mouse-GST	Proteintech	66001-2-Ig	1:5000
anti-Mouse-c-Fos	Proteintech	66590-1-Ig	1:5000
Alexa Fluor 488-anti Mouse IgG	Cell signaling technology	4408S	1:500
Alexa Fluor 488-anti Rabbit IgG	Cell signaling technology	4412S	1:500
Alexa Fluor 555-anti Mouse IgG	Cell signaling technology	4409S	1:500
Alexa Fluor 555-anti Rabbit IgG	Cell signaling technology	4413S	1:500
HRP-conjugated Goat Anti-Rabbit IgG(H+L)	Proteintech	SA00001-2	1:20000
HRP-conjugated Goat Anti-Mouse IgG(H+L)	Proteintech	SA00001-1	1:20000
