# Supplementary material for: Regulation of the PKD2 channel function and associated disease phenotypes by RASSF4
Source: Commun Biol. 2026 May 15;9:1035. doi: 10.1038/s42003-026-10242-8 (PMC13434659; doi:10.1038/s42003-026-10242-8)
Supplement: Supplementary file 2 [file 42003_2026_10242_MOESM4_ESM.pdf]

nature portfolio

Corresponding author(s): Xing-Zhen Chen , COMMSBIO-25-5535

Last updated by author(s): Jan 6, 2026

Reporting Summary

Nature Portfolio wishes to improve the reproducibility of the work that we publish. This form provides structure for consistency and transparency in reporting. For further information on Nature Portfolio policies, see our Editorial Policies and the Editorial Policy Checklist.

Statistics

For all statistical analyses, confirm that the following items are present in the figure legend, table legend, main text, or Methods section.

n/a	Confirmed	
☐	☒	The exact sample size (*n*) for each experimental group/condition, given as a discrete number and unit of measurement
☐	☒	A statement on whether measurements were taken from distinct samples or whether the same sample was measured repeatedly
☐	☒	The statistical test(s) used AND whether they are one- or two-sided<br>*Only common tests should be described solely by name; describe more complex techniques in the Methods section.*
☒	☐	A description of all covariates tested
☒	☐	A description of any assumptions or corrections, such as tests of normality and adjustment for multiple comparisons
☐	☒	A full description of the statistical parameters including central tendency (e.g. means) or other basic estimates (e.g. regression coefficient) AND variation (e.g. standard deviation) or associated estimates of uncertainty (e.g. confidence intervals)
☐	☒	For null hypothesis testing, the test statistic (e.g. *F*, *t*, *r*) with confidence intervals, effect sizes, degrees of freedom and *P* value noted<br>*Give P values as exact values whenever suitable.*
☒	☐	For Bayesian analysis, information on the choice of priors and Markov chain Monte Carlo settings
☒	☐	For hierarchical and complex designs, identification of the appropriate level for tests and full reporting of outcomes
☒	☐	Estimates of effect sizes (e.g. Cohen's *d*, Pearson's *r*), indicating how they were calculated

Our web collection on statistics for biologists contains articles on many of the points above.

Software and code

Policy information about availability of computer code

Data collection	A Geneclamp 500B amplifier, a Digidata 1322A AD/DA converter, and pClamp 10 (Molecular Devices, Union City, CA) for recording oocyte currents<br>Leica SP8 for confocal imaging<br>Leica M205 FA stereo fluorescence microscope for zebrafish imaging
Data analysis	Statistical analysis was performed using GraphPad Prism 6 (6.01 version).<br><br>Graph were generated by GraphPad Prism 6 (6.01 version).<br><br>Image processing and blot data analysis was performed using the Fiji ImageJ software (Win 64 version).

For manuscripts utilizing custom algorithms or software that are central to the research but not yet described in published literature, software must be made available to editors and reviewers. We strongly encourage code deposition in a community repository (e.g. GitHub). See the Nature Portfolio guidelines for submitting code & software for further information.

Data

Policy information about availability of data

All manuscripts must include a data availability statement. This statement should provide the following information, where applicable:

- Accession codes, unique identifiers, or web links for publicly available datasets
- A description of any restrictions on data availability
- For clinical datasets or third party data, please ensure that the statement adheres to our policy

All data supporting the findings of this study are available within the paper and its Supplementary Information.

Research involving human participants, their data, or biological material

Policy information about studies with human participants or human data. See also policy information about sex, gender (identity/presentation), and sexual orientation and race, ethnicity and racism.

Reporting on sex and gender	N/A
Reporting on race, ethnicity, or other socially relevant groupings	N/A
Population characteristics	N/A
Recruitment	N/A
Ethics oversight	N/A

Note that full information on the approval of the study protocol must also be provided in the manuscript.

Field-specific reporting

Please select the one below that is the best fit for your research. If you are not sure, read the appropriate sections before making your selection.

☒ Life sciences ☐ Behavioural & social sciences ☐ Ecological, evolutionary & environmental sciences

For a reference copy of the document with all sections, see nature.com/documents/nr-reporting-summary-flat.pdf

Life sciences study design

All studies must disclose on these points even when the disclosure is negative.

Sample size	Sample size was chosen based on the need to have sufficient statistical power.
Data exclusions	No data were excluded from the analyses.
Replication	All statistical data were obtained from at least three independent experiments from which representative data were selected.
Randomization	Randomization was used for all experimental groupings.
Blinding	The investigators were blinded to group allocation during data collection and analysis.

Reporting for specific materials, systems and methods

We require information from authors about some types of materials, experimental systems and methods used in many studies. Here, indicate whether each material, system or method listed is relevant to your study. If you are not sure if a list item applies to your research, read the appropriate section before selecting a response.

Materials & experimental systems

n/a	Involved in the study	
☐	☒	Antibodies
☐	☒	Eukaryotic cell lines
☒	☐	Palaeontology and archaeology
☐	☒	Animals and other organisms
☒	☐	Clinical data
☒	☐	Dual use research of concern
☒	☐	Plants

Methods

n/a	Involved in the study	
☒	☐	ChIP-seq
☐	☒	Flow cytometry
☒	☐	MRI-based neuroimaging

Antibodies

Antibodies used

Antibodies used in western blots, co-immunoprecipitation and immunostaining were listed Table 1 in the manuscript. Antibodies for western blot used at a dilution of 1:500~1:5,000. All antibodies used for immunostaining were at a dilution of 1:500.

Validation

All antibodies in the study were bought commercially and the corresponding validation information is available on the manufacturers' websites:
anti-PKD2(Proteintech,19126-1-AP;1:2000)
https://www.ptgcn.com/products/PKD2-Antibody-19126-1-AP.htm
Species specifity: Rabbit
Application: WB, IHC, IP, ELISA
Publication: PMID: 38254271
anti-RASSF4(Thermo Fisher,1PA5-103245;1:2000)
https://www.thermofisher.cn/cn/zh/antibody/product/RASSF4-Antibody-Polyclonal/PA5-103245
Species specifity: Rabbit
Application: WB, ICC,IF
Publication: not published
anti-GAPDH(Proteintech, 60004-1-Ig;1:5000)
https://www.ptgcn.com/products/GAPDH-Antibody-60004-1-Ig.htm
Species specifity: Mouse
Application: WB, IHC, IF/ICC, FC
Publication:PMID: 36477534
anti- β-actinS anta cruz,sc-47778 ;1:5000)
https://www.ptgcn.com/products/GAPDH-Antibody-60004-1-Ig.htm
Species specificity: Mouse
Application: WB, IP, IF, IHC (P) and ELISA
Publication:PMID: 39940667
anti-HA (Sima,05904;1:2000)
https://www.sigmaaldrich.cn/CN/zh/product/mm/05904
Species specificity: Mouse
Application: WB, IP, ICC
Publication: PMID: 24733071
anti-HA (Proteintech,51064-2-AP;1:5000)
https://www.ptgcn.com/products/HA-tag-Antibody-51064-2-AP.htm
Species specificity: Rabbit
Application: WB, IF/ICC, FC (Intra), IP, ELISA
Publication: PMID: 38575583
anti- DYKDDDDK (MBL,M185-3S;1:5000)
https://www.mbl-chinawide.cn/search012?keyword=M185-3S
Species specificity: Mouse
Application: WB, IF/ICC, FC (Intra), IP, ELISA
Publication: PMID: 28333142
anti- DYKDDDDK (Proteintech,20543-1-AP;1:5000)
https://www.ptgcn.com/products/Flag-Tag-Antibody-20543-1-AP.htm
Species specificity: Rabbit
Application: WB, IF/ICC, IP, ELISA
Publication: PMID:34739333
anti- ERK (CST,4695T;1:1000)
https://www.cellsignal.cn/products/primary-antibodies/p44-42-mapk-erk1-2-137f5-rabbit-monoclonal-antibody/4695
Species specificity: Rabbit
Application: WB, IF/ICC, IP
anti- p-ERK (CST,4377T;1:1000)
https://www.cellsignal.cn/products/primary-antibodies/phospho-p44-42-mapk-erk1-2-thr202-tyr204-197g2-rabbit-monoclonal-antibody/4377
Species specificity: Rabbit
Application: WB, IF
Publication: PMID: 15187187
anti- GFP (Proteintech,50430-2-AP;1:2000)
https://www.ptgcn.com/products/eGFP-Antibody-50430-2-AP.htm
Species specificity: Rabbit

Application: WB, IF/ICC, IF-P, IP, ELISA
Publication: PMID: 38168988
anti- GFP (Proteintech,66002-1-Ig;1:2000)
https://www.ptgcn.com/products/eGFP-Antibody-66002-1-Ig.htm
Species specificity: Mouse
Application: WB, IF/ICC, IP, ELISA
Publication: PMID: 39013469
anti- GST (Proteintech,66001-2-Ig;1:5000)
https://www.ptgcn.com/products/eGFP-Antibody-66002-1-Ig.htm
Species specificity: Mouse
Application: WB, IP, ELISA
Publication: PMID: 39251763
anti- GST (Proteintech,66590-1-Ig;1:5000)
https://www.ptgcn.com/products/FOS-Antibody-66590-1-Ig.htm
Species specificity: Mouse
Application: WB, FC (Intra), ELISA
Publication: PMID: 40434197

Eukaryotic cell lines

Policy information about cell lines and Sex and Gender in Research

Cell line source(s)	HEK293T: ATCC CRL3216: MDCK: ATCC CRL-2936; U-2OS: ATCC HTB-96.
Authentication	All the cells were authenticated using short-tandem repeat profiling.
Mycoplasma contamination	All cell lines were tested negative for mycoplasma contamination by PCR.
Commonly misidentified lines (See ICLAC register)	No commonly misidentified cell line was used in the study.

Animals and other research organisms

Policy information about studies involving animals; ARRIVE guidelines recommended for reporting animal research, and Sex and Gender in Research

Laboratory animals	Zebrafish AB line (ZFIN ID: ZDB-GENO-960809), female or male aged 3-4 month, were used for crossing. All zebrafish were cultured and bred at 28°C.<br>Xenopus laevis oocytes were obtained from adult female frogs. All frogs were maintained in a recirculating water system at a constant temperature of 18–20°C. Ovarian lobes were surgically removed from females anesthetized on ice, and stage V–VI oocytes were isolated using fine forceps. The oocytes were subsequently cultured in an incubator at 18°C for microinjection or electrophysiological recordings.
Wild animals	N/A
Reporting on sex	We used zebrafish larvae as a disease model and performed imaging at the early larval stage. For experimental design considerations, the mice used in this study were male. The male mice employed in the experiments were procured, and their sex was confirmed by measuring the anogenital distance.
Field-collected samples	N/A
Ethics oversight	All mouse and zebrafish procedures were conducted under the guideline and the institutional animal care protocol approved by the experimental animal committee at Hubei University of Technology. All experimental procedures involving Xenopus oocytes were conducted in accordance with the institutional animal care guidelines and protocols approved by the Animal Care Committee of the University of Alberta.

Note that full information on the approval of the study protocol must also be provided in the manuscript.

Plants

Seed stocks	N/A
Novel plant genotypes	N/A
Authentication	N/A

Flow Cytometry

Plots

Confirm that:

☒ The axis labels state the marker and fluorochrome used (e.g. CD4-FITC).

☒ The axis scales are clearly visible. Include numbers along axes only for bottom left plot of group (a 'group' is an analysis of identical markers).

☒ All plots are contour plots with outliers or pseudocolor plots.

☒ A numerical value for number of cells or percentage (with statistics) is provided.

Methodology

Sample preparation	MDCK cells to be collected were washed and digested with trypsin to prepare a single-cell suspension. Propidium iodide (PI) staining solution was then added, followed by incubation for 10 minutes under light-protected conditions, in preparation for flow cytometric analysis.
Instrument	Data were acquired on a Sony SH800S Series cell sorter (Sony Biotechnology).
Software	Data were acquired using the native Sony Software (v1.4.1) and analyzed with ModFitLT (v3.1).
Cell population abundance	Following PI staining and analysis of MDCK cells, the proportion of cells in the G1 phase was 50% ± 5% (mean ± SD, n=3). The reliability of the cell cycle distribution was assessed by evaluating the goodness of fit of the DNA content histograms, and the coefficient of determination (R^2) for the cell cycle model used in all cases exceeded 0.95.
Gating strategy	For cell cycle analysis using PI staining, the following gating and analysis strategy was employed. First, unstained cells were used as a negative control to set the baseline. Cells were initially gated on an FSC-A versus SSC-A scatter plot to exclude small debris and large aggregates. Single cells were then selected using an FSC-H versus FSC-A plot to remove any remaining doublets or cell clusters. To specifically analyze the DNA content of intact nuclei, cells were gated based on a tight diploid DNA content distribution on a linear scale in the PI fluorescence channel. The boundaries defining the G0/G1, S, and G2/M phase populations were determined by applying a cell cycle fitting model to the DNA content histogram of this gated single-cell population. The goodness of fit of the model and the accuracy of phase assignment were verified by a low root mean square error (RMSE) and a coefficient of determination (R^2) greater than 0.95.

☐ Tick this box to confirm that a figure exemplifying the gating strategy is provided in the Supplementary Information.
