# Supplementary material for: Regulation of the PKD2 channel function and associated disease phenotypes by RASSF4
Source: Commun Biol. 2026 May 15;9:1035. doi: 10.1038/s42003-026-10242-8 (PMC13434659; doi:10.1038/s42003-026-10242-8)
Supplement: Supplementary file 3 [file 42003_2026_10242_MOESM2_ESM.docx]

**Description of Additional Supplementary File**

File name: Supplementary Data

Description: The original data for the graphs and tables.
